# Recent Advances in the Application of Coumarins as Photosensitizers for the Construction of a Dye-Sensitized Solar Cell

**DOI:** 10.1021/acsomega.4c11135

**Published:** 2025-04-01

**Authors:** Edson Evangelista, Iva S. de Jesus, Fernanda P. Pauli, Acácio S. de Souza, Amanda de A. Borges, Maria Vitória S. F. Gomes, Vitor F. Ferreira, Fernando de C. da Silva, Mauricio A. Melo, Luana da S. M. Forezi

**Affiliations:** † Departamento de Química Orgânica, Universidade Federal Fluminense - UFF, Niterói, Rio de Janeiro 24020-141, Brazil; ‡ Programa de Pós-Graduação em Química - UFF, Niterói, Rio de Janeiro 24020-141, Brazil; § Departamento de Tecnologia Farmacêutica, Universidade Federal Fluminense - UFF, Niterói, Rio de Janeiro 24241-000, Brazil; ∥ Programa de Pós-Graduação em Ciências Aplicadas a Produtos para a Saúde - UFF, Niterói, Rio de Janeiro 24241-000, Brazil; ⊥ Departamento de Química Inorgânica, Universidade Federal Fluminense - UFF, Niterói, Rio de Janeiro 24020-141, Brazil

## Abstract

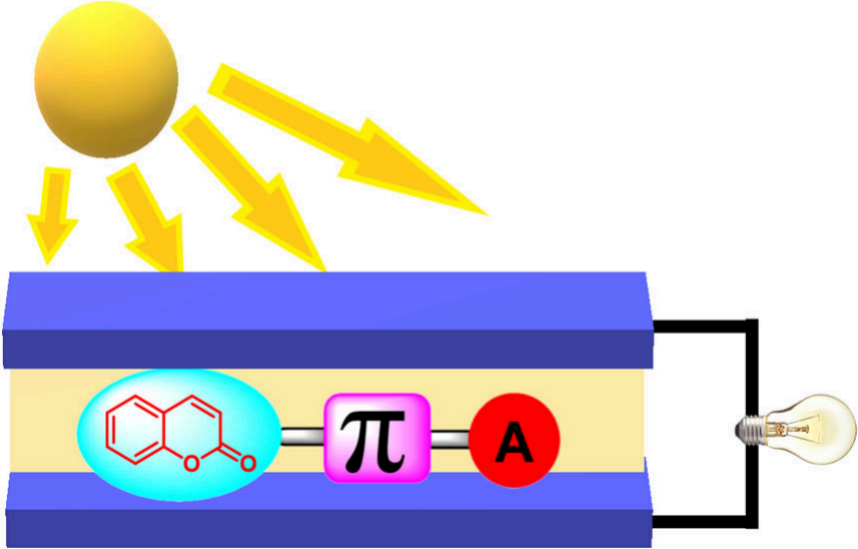

Dye-sensitized solar cells (DSSCs) have emerged as a promising alternative in photovoltaic energy, owing to their efficiency, cost-effectiveness, and versatility. Sensitizers are crucial in efficiently converting solar energy into electricity in these systems. This review focuses on employing coumarins, a class of aromatic organic compounds, as sensitizers in DSSCs. We address the synthesis of coumarins, their photophysical and electronic properties, and their application in optimizing the energy conversion efficiency of DSSCs. We explore the molecular engineering strategies used to improve the properties of coumarins, including structural modification and the combination with other materials. Furthermore, we discuss current challenges and prospects for the development of DSSCs using coumarins as sensitizers. This review aims to provide a comprehensive overview of the current state and future directions of this exciting area of solar energy research.

## 1 Introduction

Due to population growth and environmental pollution, clean energy has become a global priority.^1^ Solar energy stands out as an abundant and sustainable source, offering a promising alternative to traditional energy resources. With a power of approximately 1.8 × 10^11^ MW coming from the Sun and being intercepted by the Earth, solar energy appears to be a viable and renewable option. Its use, mainly through photovoltaic (PV) technology, allows the direct conversion of solar radiation into electricity, proving to be an efficient tool to take advantage of this resource.^1,2^

Recently, dye-sensitized solar cells (DSSCs) have emerged as a promising alternative in energy generation notable for their economic accessibility, efficiency, and unlimited potential.^3−5^ The DSSCs represent an emerging technology, with increasing efficiencies and performance longevity, competing with conventional silicon-based technologies.^3,5−7^ The diversity of organic molecules that can be applied in the construction of DSSCs offers an additional advantage, enabling the formulation of varied donors, acceptors, and interfaces. DSSCs offer low cost, ecological compatibility, and acceptable efficiency compared to traditional silicon cells.

In recent studies, coumarin-based DSSCs have aroused great interest due to their ability to convert visible light into electrical energy more efficiently.^8,9^ The photosensitizer, a crucial component of these cells, plays a fundamental role in their performance. However, the search for ideal components for DSSCs is a costly and time-consuming process.^6,7^ Thus, quantum chemical methods combined with efficient software packages become vital to accelerate the discovery and optimization of new materials for DSSCs, boosting their efficiency and applicability.^10^ In this context, this review article covers the most recent advances and the performance of photosensitizers that include coumarins in their structures or that act as cosensitizers, aiming to increase the efficiency of DSSCs.

### 1.1 Dye-Sensitized Solar Cells

DSSCs represent an innovation in the field of energy generation, distinguished from conventional photovoltaic cells by using two distinct materials for specific functions: charge transport and photon absorption.^11−13^ The essential components of a DSSC are the photoelectrode (anode), the counter electrode (cathode), and the electrolyte, with the photoanode consisting of a conductive substrate covered with fluorine-doped tin oxide (FTO) and a nanocrystalline metal oxide film, generally TiO_2_.^14,15^

**Figure 1:**
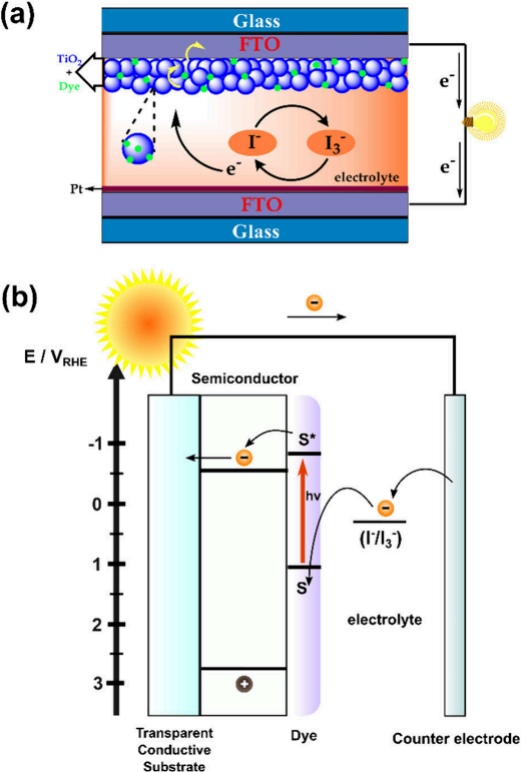
Schematic illustrations of (a) the operation of a dye-sensitized cell and (b) the energy diagram depicting excitation and charge transfers in DSSCs.

The fundamental principle of DSSCs involves a nanocrystalline TiO_2_ electrode film deposited on a transparent conductive oxide substrate, sensitized with a dye.^12,14^ This configuration also includes a Pt counter electrode and an electrolyte containing iodide/tri-iodide ions, essential to restoring the dye to its original state, acting as a redox couple (Figure 1a).

In the operation of DSSCs, the first step is the generation of photoexcited electrons when incident photons are absorbed by the dye, which acts as an electron-generating layer. This step involves the excitation of the dye grafted onto the electrode (S → S*). Electrons are then injected from the excited dye into the conduction band (CB) of the semiconductor (usually TiO_2_), leaving the dye molecule in an oxidized state. TiO_2_, in turn, acts as a transport layer in which these electrons diffuse, governed by a chemical gradient.^14^ After the transfer of photoexcited electrons to the CB of TiO_2_, they are collected at the transparent conductive electrode and then transferred to the external circuit. Through the external circuit, the electrons reach the counter-electrode. In addition to TiO_2_, various metal oxides and composite materials like Au-TiO_2_,^16^ ZnO,^17,18^ Ag_2_0-ZnO,^19^ Nb_2_O_5_,^20^ SnO_2_,^21^ WO_3_,^22^ and Fe_2_O_3_^23,24^ are subject to investigation as potential photoanode materials.^25^ After reaching the counter-electrode, the electrons are transferred to the oxidant of the redox pair dissolved in the electrolyte, which assumes the reduced form. Under this reduced form, the redox mediator reduces the oxidized dye, thus completing the cycle necessary for generating electrical energy from incident light.^26−28^ The I/I_3_ mixture is generally used as a redox couple, and other alternative redox pairs, including pseudohalogen-based redox pairs,^29−31^ organic redox pairs,^32−41^ ferricenium/ferrocene,^42,43^ and metal complex redox pairs,^44−47^ were investigated to replace I/I_3_.^48^ All of the steps involving the solar-light excitation, charge transfer, and redox processes are depicted in the energy diagram of Figure 1b.^13^

The pioneering work focused on developing DSSCs dates back to the work of O’Regan and Grätzel in 1991, which achieved an efficiency of 7.9%.^8,14,15,49^ Since then, the evolution of these devices, especially with the application of nanotechnology, has enabled significant improvements. The use of metal oxide nanoparticles has been researched in nanocrystalline and mesoporous films, with a large surface area for the adsorption of substantial amounts of dye, boosting the efficiency of these cells.^11,15^

The presence of a sensitizer is indispensable since TiO_2_ does not absorb the visible region of solar radiation. Furthermore, the counter electrode, covered with a thin deflection layer, such as platinum or graphite nanoparticles, completes the sandwich arrangement. A redox couple is typically introduced between these components to regenerate the dye and facilitate proper functioning of the DSSCs. Although numerous types of redox couples are described in the literature, the I/I_3_ combination is the most used in the works described.^50−53^ Several factors determine the efficiency of solar cells, but the structural and physical properties of sensitizers are among the most important ones.^54^

In the past 25 years, a remarkable journey has unfolded in the development of donor and acceptor materials.^55^ Continuous breakthroughs have ushered in high-performance variants, progressively enriching the landscape field of DSSCs. The performance metrics focus predominantly on the short-circuit current density (*J*_SC_), open-circuit voltage (*V*_oc_), fill factor (FF), and photoconversion efficiency (PCE or η). The relationship between these parameters is represented by the following equation^3,7,54,56^
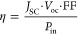
where *P*_in_ is a constant associated with the incident light.

Enhancing the photovoltaic capabilities of DSSCs inherently involves the strategic optimization of these parameters. Achieving maximum η crucially hinges on augmenting the electronic density originating from electronic donor groups housed within the HOMO. Moreover, expanding the conjugated π-bridge assumes essential importance, orchestrating a shift in energy levels between HOMO and LUMO.^55^ This strategic maneuver aims to realize a more efficient intramolecular charge separation, thereby elevating the overall performance of the DSSCs.

Among the various structural possibilities for sensitizers, ruthenium dyes have historically been the most used in DSSCs due to their excellent light absorption capacity, stability, and efficiency in electron injection. DSSCs based on ruthenium dyes, such as N719, have achieved remarkable efficiencies. For example, DSSCs with polymer gel electrolytes (PGEs) based on PVDF-HFP and ruthenium dyes have reached efficiencies of up to 8.97% under 1 sun illumination (AM 1.5G, 100 mW/cm^2^).^57^ Furthermore, combining ruthenium dyes with polymer gel electrolytes has demonstrated good long-term stability, retaining 90% of the initial efficiency after 1000 h of testing under high light and temperature conditions. The use of gelled electrolytes can mitigate sealing and leakage issues associated with liquid electrolytes, enhancing the safety and flexibility of the devices. Dye-sensitized solar cells (DSSCs) based on organic D-π-A dyes, such as MD-153, have achieved an impressive efficiency of 10.1% when combined with PVDF-HFP gel electrolytes and the redox mediator TEMPO/TEMPO+. These dyes have also demonstrated high efficiency under ambient light conditions, with efficiencies exceeding 25% under 1000 l× illumination (compact fluorescent light).^57−61^ Furthermore, research on alternative redox systems, such as the use of copper-based mediators, has shown promising efficiency and stability. The flexibility of D-π-A dyes and the ability to optimize gelled electrolytes are factors that contribute to the commercial viability of these DSSCs in indoor applications and low-power electronic devices.^58^

Another possible structure for application in DSSCs is porphyrin dyes, which are known for their strong absorption in the visible region of the electromagnetic spectrum and their ability to generate multiple excited states, making them promising candidates for DSSCs.^61^ Porphyrin dyes, such as CYC-B11, have been used in combination with polymer-gel electrolytes, achieving efficiencies of up to 10.58% under 1 sun illumination. Furthermore, the stability of these devices has been tested, with a retention of 75% of the initial efficiency after 700 h of continuous testing under high light and temperature conditions. The research also highlights the importance of alternative redox mediators, such as iron-based systems, which have shown encouraging results in terms of efficiency and stability. The use of gelled electrolytes in porphyrin DSSCs can improve efficiency and durability, making them more competitive compared to solid photovoltaic technologies.^60,61^ In summary, the convergence of advancements in DSSCs emphasizes the critical role of materials and strategies in reshaping the photovoltaic landscape. The evolution of these materials and the strategic optimization of pertinent parameters remain instrumental in propelling the efficiency and efficacy of solar cell technologies toward unprecedented horizons.

### 1.2 Coumarins

**Figure 2:**
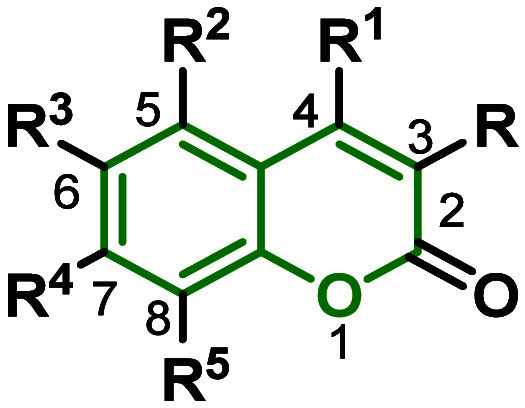
General structure of coumarins and their functionalizable sites.

Coumarins, aromatic lactones primarily found in plants, initially lack luminescence. However, by introducing an electron-donating group at position 7, the compound exhibits significant light absorption and luminescence, as depicted in Figure 2.^62^

Coumarins have attracted significant interest in recent years due to their photophysical properties. Widely employed as fluorescent probes and components in organic light-emitting diodes (LEDs), they stand out for their remarkable ability to modify color and fluorescence through the selective introduction of substituents.^63,64^ Electron-donating groups at position 7 and electron-withdrawing groups at position 4 or 3 are essential to enhancing intramolecular charge transfer (ICT) in these molecules. The inclusion of groups such as ethynyl, thiophenyl, and styryl expands the conjugated system, altering the energy levels of frontier orbitals and increasing the molar absorptivity of coumarins.^65^

Coumarins do not exhibit luminescence until an electron donor is added at position 7, initiating light absorption and luminescence through ICT between electron donors and acceptors.^62^ Manipulating the wavelength in absorption and luminescence spectra is possible by adding groups at position 3 or 4. Connecting an electron-withdrawing group at these positions can intensify luminescence.^65^ The influence of different groups on the luminescence and fluorescence of coumarins can be evidenced in several works reported in the literature;^66−70^ however, in a recent work, Sekine and colleagues^69^ demonstrate how different donor groups in position 7 and other substituents in positions 3 and 4 affect their photophysical properties. In the research, the donation capacity of the substituent in position 7 and the interference in the conjugation of the structure when having a substituent in position 3 or 4 is observed, so the results interfere with such groups as evident when analyzing different analogs and proving what was reported until then (Table 1).

**Table 1:**
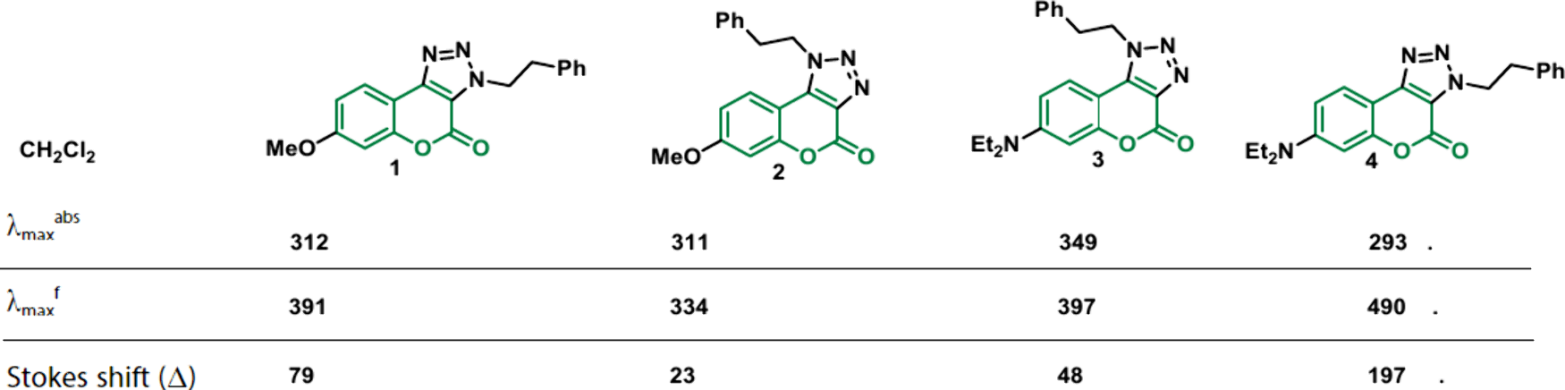
Fluorescence Properties of Triazolo[c]coumarins

These molecules play a central role in the development of DSSCs, being effective in light absorption and solar conversion to electrical energy.^62^ Achieving optimal efficiencies and prolonging the lifespan of these cells are top priorities.^71^ Understanding parameters governing their efficiency, such as the molecular structure and HOMO–LUMO levels, is essential. Studying the kinetics and dynamics of charge movement in this system is crucial. Characteristics such as light absorption, the maximum absorption wavelength, and the energy gap of dye materials are crucial in DSSC development.^72,73^

Coumarin-based dyes have been effectively utilized in DSSCs, yielding photovoltaic conversion efficiencies of up to around 8%. They demonstrate great photoresponse in the visible region, long-haul dependence under exposure, and appropriate energy-level arrangement for injection into the CB of TiO_2_.^73^ Coumarins remain an exceptionally intriguing class of compounds, given the rapid injection rates observed for TiO_2_ substrates and the accessibility of a vast scope of synthetic derivatives that display particularly unique properties.^72,73^

## 2 Studies as a Donor Group

In DSSCs, the donor group plays a key role in influencing photovoltaic performance.^74^ A good donor group results from an excellent light absorption spectrum and energy levels greater than the CB of the metal oxide semiconductor (usually TiO_2_) as well as the redox potential of the electrolyte (usually, I^–^/I^–3^). Therefore, donor selection and structural optimization are crucial for the spectral response, efficient charge injection, and stability of sensitizers with high photovoltaic performance. To develop metal-free organic sensitizing dyes with favorable photophysical properties and energy levels suitable for both the photoanode and electrolyte, several electron donors, such as triarylamine,^75,76^ indoline,^77,78^ diphenylamine, carbazole, phenothiazine, and coumarin,^79,80^ have been explored as essential components that contribute to injecting electrons into the acceptor due to their electron-donating capabilities and good thermal and electrochemical stability, thus determining the absorption spectra of the sensitizers. Among the mentioned nuclei, coumarins stand out for their high functional ability, allowing for the incorporation of diverse donor groups within the same molecule. This versatility opens up a spectrum of possibilities, making coumarins excellent candidates for exploration as donor groups in DSSC sensitizers.^81−83^

**Scheme 1:**
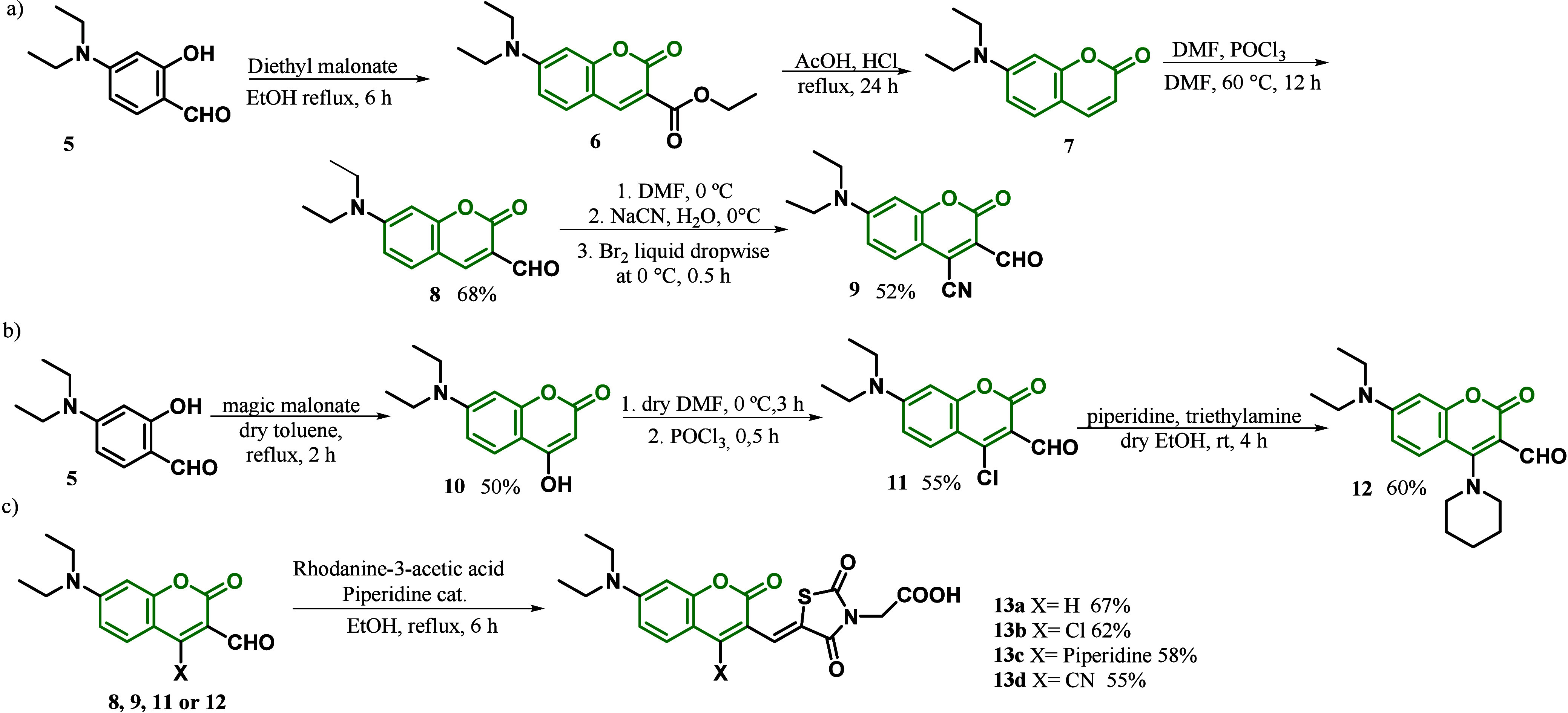
a) Synthesis of Intermediates **8** and **9**, b) Synthesis of Intermediates **11** and **12**, and c) Synthesis of Coumarin-Derived Dyes **13a**–**d**

In 2018, Jadhav and co-workers^84^ related the synthesis and the evaluation of the donor effect of newly 4-substituted donor-π-acceptor coumarins. It was observed that the photophysical and photovoltaic properties of the sensitizer in converting light to current are sensitive to substitution at position 4 due to the change in the HOMO–LUMO band gap, with the electron density distribution between these levels also being affected. The synthesis of coumarin dyes **13a**–**d** is shown in Scheme 1. Compound **8** was obtained from the condensation reaction of 4-(diethylamino)-2-hydroxybenzaldehyde **5** with diethyl malonate to furnish **6**, which was then treated with DMF in POCl_3_. The substitution reaction between **8** and sodium cyanide, mediated by bromine, gave compound **9** as the product. Compound **11** was obtained by the condensation reaction between bis(2,4,6-trichlorophenyl)malonate^85^ and **5**, generating **10**, which was treated with POCl_3_. Compound **12** was obtained through a substitution reaction between molecules of **11**, and piperidine-type **13** dyes were obtained via Knoevenagel condensation between the rhodanine-3-acetic acid and the respective coumarin derivatives **8**, **9**, **11**, and **12** in 55–67% yields.

Compound **13a** was used as a reference for comparison to other dyes. In coumarin dyes **13b** and **13c**, the effects caused by the electronic and steric properties of the substituents present at the C4 position of the coumarin ring were evaluated. The electron density on the chlorine or nitrogen atom for **13b** or **13c** suggests its importance as a primary donor, while the diethylamine group acts as an auxiliary donor. The photovoltaic devices based on **13a**–**c** as sensitizers were measured under 1.5 AM solar irradiation (100 mW·cm^–2^). The photophysical properties, cyclic voltammetry characterization, and density functional theory calculation of **13a**–**c** suggest the potential use of these dyes as sensitizers for DSSCs. **13a** showed a lower efficiency of 2.64%, which was attributed to the low *J*_SC_ (6.85 mA). Chlorine in **13b** acts as an electron donor, increasing the efficiency to 3.19%. For **13c**, position 4 is replaced by the piperidine group, with an efficiency of 3.79% due to the increase in the short-circuit current (8.62 mA), showing that the group donor in position 4 increases cell efficiency. From the comparison, it is evident that the introduction of the substituent in position 4 increases the efficiency of the devices, significantly improving *J*_SC_, which is due to the better charge separation and ease of injection of electrons from the sensitizer into the CB of TiO_2_.^84^

**Scheme 2:**
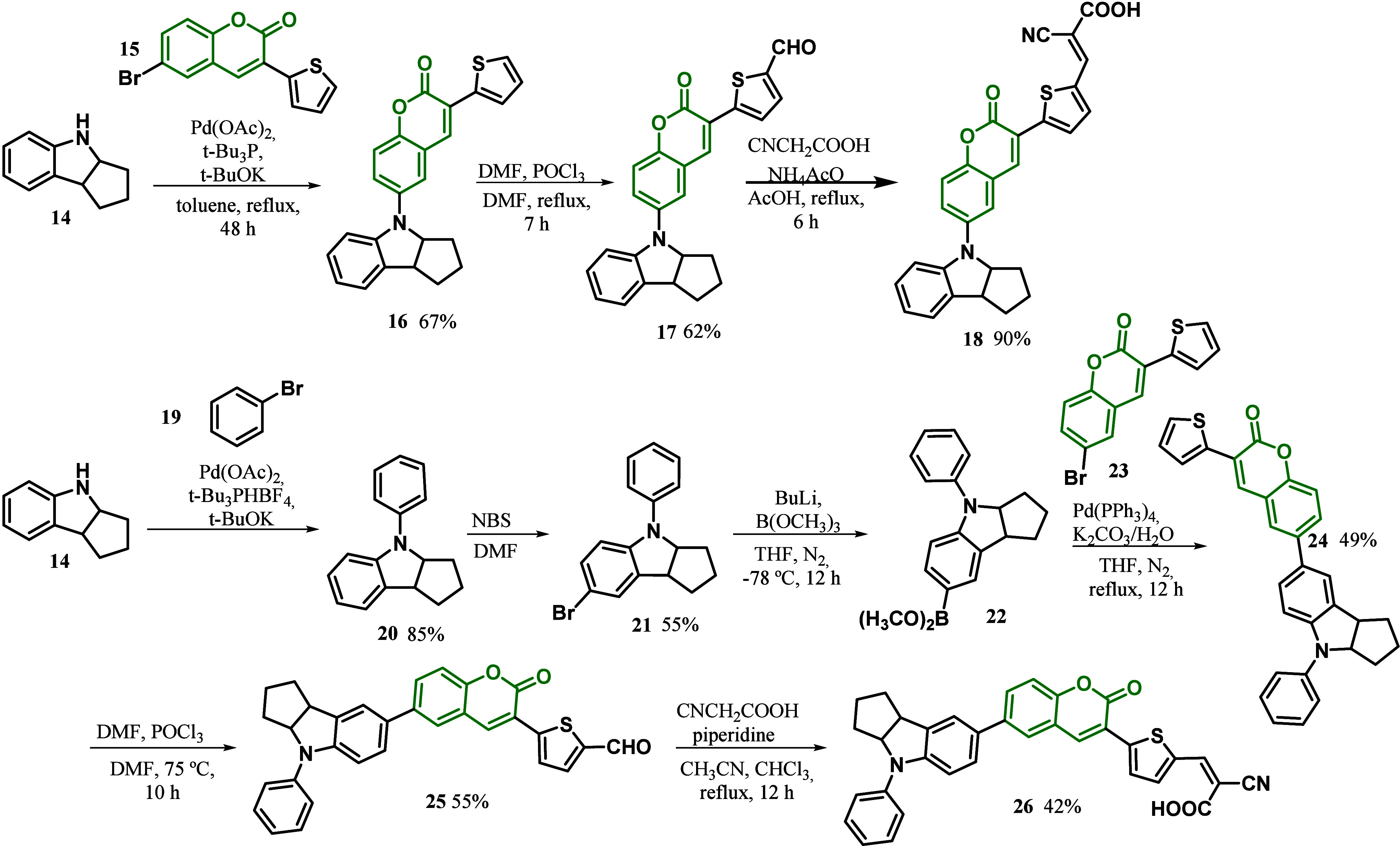
Synthesis of the Indole-Linked Sensitized Coumarin Dye

In 2019, Jiang and co-workers^86^ reported the synthesis of novel D-D-π-A sensitizers containing two new indole-linked dye-sensitized coumarins. Electron-rich chromophores, such as coumarin derivatives, are used as electron donors, characterized by good photoresponse in the visible region. The introduction of an additional donor in the reported D-D-π-A sensitizers, such as indole, may expand the light absorption region, increase the molar extinction coefficient, and reduce aggregation, which benefits the light-harvesting ability and electron injection efficiency. The synthetic pathway of indoline-linked coumarin dye is depicted in Scheme 2. Bromobenzene was introduced into **14** under the catalysis of Pd(OAc)_2_ to give *N*-phenylindoline **20**. After bromination, **21** was transformed into boronate **22** with B(OMe)_3_. Boronate **22** reacted directly with 6-bromo-3-thiophenylcoumarin **23** through a Suzuki coupling reaction to synthesize compound **24**. The aldehyde group was introduced into the thiophene ring of **24** through Vilsmeier reagent to afford intermediate aldehyde **25**. Dye **26** was prepared through the Knoevenagel condensation of **25** and cyanoacetic acid. On the other hand, 6-bromo-3-thiophenylcoumarin reacted directly with indole **14** to afford coupling product **16**. Compound **16** was transformed into aldehyde **17**, which further condensed with cyanoacetic acid to give dye **18**.

Density functional theory (DFT) studies have shown that dye **24** exhibits good planarity, with the coumarin donor being in the same plane as the thiophene bridge and the cyanoacetic acid acceptor. The incorporation of the indole group, replacing the diethylamine group normally used in these dyes, results in a large dihedral angle between the indole and coumarin substructures, which deteriorates the total molecular coplanarity of dyes **18** and **26**. As a result, the weakening of intramolecular charge transfer increases the transition energy and, consequently, the blue-shifting of the absorption peaks of dyes **18** and **26**. The different binding modes between indole and coumarin also generate different electron distributions in the boundary molecular orbitals in dyes **18** and **26**, especially in HOMO. While HOMO do **18** is distributed throughout the structure, HOMO do **26** is mostly delocalized over the indole donor subunit and partially spread over the benzene ring of coumarin. Both LUMOs are mainly composed of the coumarin moiety, π-bridge, and acceptor. The good overlap between the HOMO and LUMO orbitals of these substances benefits the efficient excitation of electrons generated by light and the subsequent injection of electrons into the TiO_2_ conduction band. The photovoltaic performance of **18**, **24**, and **26** dye-sensitized DSSCs was measured under AM 1.5 G solar light. **26**-sensitized DSSC achieved a higher *J*_SC_ (11.09 mA·cm^–2^) than that of **24**-sensitized DSSC (8.68 mA·cm^–2^) and **18**-sensitized DSSC (7.51 mA·cm^–2^). In the case of **18**, a less intense absorption spectrum indicates its low light-harvesting efficiency, which leads to an unsatisfactory incident photo-to-current conversion efficiency. Thus, **18**-sensitized DSSCs show the smallest *J*_SC_. *V*_oc_ has been improved distinctly through replacement of the diethylamino group with the indoline group. The theoretical calculation and EIS results showed that the incorporation of the indoline group decreases the coplanarity of **26** and **18**, which lowers their charge recombination rate and improves the *V*_oc_ of **26** and **18** to above 0.7 V from 0.57 V of **24**. Therefore, among the three dyes, **26**-sensitized solar cells achieved an optimal photovoltaic conversion efficiency of 5.47% derived from its better *J*_SC_ (11.09 mA·cm^–2^) and *V*_oc_ (0.72 V), which further suggests that the indoline group on the coumarin donor ring benefits the improvement of photovoltaic performance by increasing *V*_oc_.^86^

**Scheme 3:**
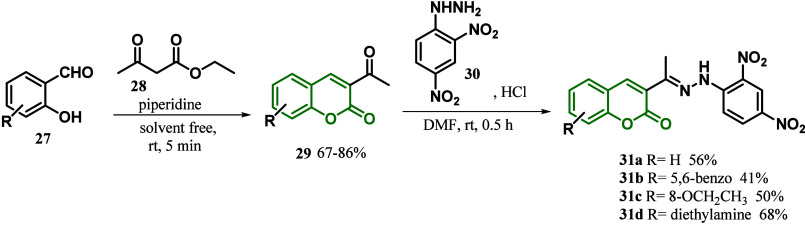
Synthesis of Coumarins **31a**–**d**

In the same year, Basavarajappa and co-workers^87^ described the synthesis and current–voltage characteristics of a series of coumarin dyes **31** that had a common acceptor and π-spacer as well as different donor substituents present at the coumarin. The synthesis of 3-acetyl coumarins **29** occurred via Knoevenagel condensation of substituted salicylaldehyde **27** with acetyl acetoacetate **28** to furnish 67–86% yields. 3-Acetyl coumarins **29** were treated with 2,4-dinitrophenylhydrazine **30**, and after a condensation reaction, the 2,4-dinitrophenylhydrazones **31a**–**d** were obtained in 41–68% yields (Scheme 3). The results reveal that an electron-releasing group helps in tuning the band gap, and the current–voltage characteristics showed a good photocurrent response under illumination conditions. The energy levels of HOMO and LUMO of the prepared dye molecules were found to be matched with TiO_2_ energy levels. Through the experimental and theoretical results, it was possible to establish a structure–property relationship between the donor and acceptor subunits and the properties of dyes **31a**–**d**. Global chemical parameters like the ionization energy (*I*), electron affinity (*A*), and electronegativity (χ) have been calculated using the values of HOMO and LUMO by FMO analysis for an understanding of donor–acceptor interactions. The ionization energy and electron affinity parameters determine the ability to donate and accept the electrons by the molecules, respectively. A smaller ionization energy makes this molecule a strong electron donor. The ionization energy is found to be lower for **31d**. This may be attributed to the presence of strong electron-releasing group diethyl amine on the 7-position of the coumarin subunit. The order of decrease in the electron affinity values for a series of dyes is as follows: **31a** > **31c** > **31b** > **31d**. The smaller electronegativity of **31d** also indicates that **31d** is a strong electron donor compared to others. The electronegativity of **31d** is greater than that of **31d** but smaller than those of **31a** and **31c**. This may be explained on the basis of the extended conjugation of the coumarin moiety with benzene ring fusion at the 5,6-position. DFT calculation of the global chemical parameters computed theoretically follows the same trend as that of experimental values, except for the electron affinity values of **31b** and **31c**. The order of the electron affinity values is as follows: **31a** > **31b** > **31c** > **31d**. The photosensitive behavior of dyes **31a**–**d** on TiO_2_ nanoparticles, in the current–voltage evaluation, is higher under light conditions compared to dark conditions for all of the dyes. The synthesized dyes were subjected to cyclic voltammetric studies in DMF to determine their electrochemical properties. The onset oxidation potentials of dyes **31a**, **31b**, **31c**, and **31d** were measured as 0.3727 0.2873, 0.3915, and 0.1788 V, respectively. From the calculations of global chemical parameters shown in Table 2, it was possible to observe that the ionization energy is lower for **31d** compared to those for the other structures, making this molecule a strong electron donor. The decreasing order of electron affinity values for the dye series is as follows: **31a** > **31b** > **31c** > **31d**. The lower electronegativity of **31d** also indicates that it is a strong electron donor compared to others. The electronegativity of **31b** is higher than that of **31d** but lower than that of **31a** and **31c**. This can be explained on the basis of prolonged conjugation of the coumarin portion with the fusion of the benzene ring at positions 5 and 6, showing that the electron donation of **31b** is greater than that of **31a** and **31c**. The hard and soft nature of the molecules can be evaluated based on the hardness and softness values.^87^

**Table 2:** Experimental Global Chemical Parameters of Synthesized Molecules

**Entry**	***I* (eV)**	***A* (eV)**	**χ (eV)**	**η (eV)**	**σ (eV)**
**31a**	5.47	3.08	4.30	1.20	0.83
**31b**	5.39	2.97	4.18	1.21	0.83
**31c**	5.49	3.08	4.29	1.21	0.83
**31d**	5.28	3.05	4.17	1.12	0.89

**Scheme 4:**
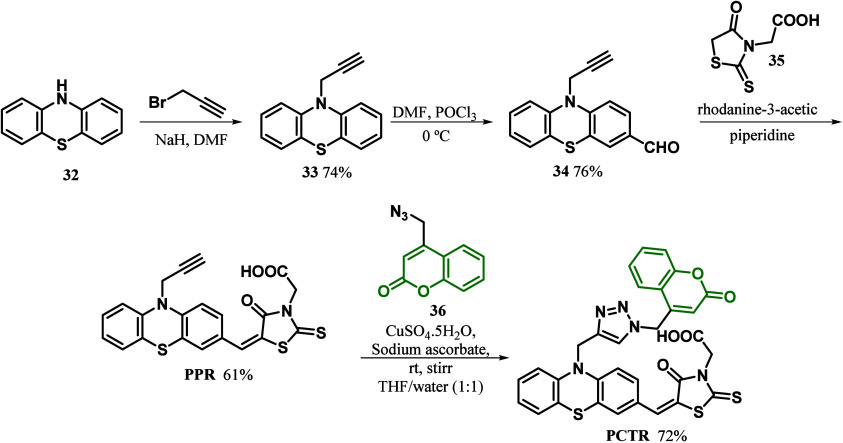
PPR and PCTR Coumarin-Based Dyes Synthesis

More recently, Mavazzan and co-workers^88^ described the development of novel metal-free organic dyes (PPR and PCTR) through a multistep synthetic route. The designed novel dye molecules were integrated into 1-D CdS nanowires, serving as an excellent sensitizer for efficient light energy harvesting components in the context of DSSCs. The dyes PPR and PCTR were synthesized in four steps (Scheme 4). In the first step, the nucleophilic substitution reaction between propargyl bromide and phenothiazide **32** produced **33**. Compound **34** was formed by further formylation of **33** with the Vilsmeier–Haack reaction. Through the reaction of **34** with rhodanine-3-acetic **35**, PPR was obtained, and PCTR, in turn, was obtained by the click chemistry approach of PPR and azide **36**.

DFT and molecular electrostatic potential (MEP) studies were employed to optimize structures and evaluate the electron distribution in the PPR and PCTR dyes. The positioning of electrons is in the HOMO level of the phenothiazine substructure and neighboring π-linkers, while electrons in the LUMO level are scattered among the neighboring π-linkers and rhodanine of PPR as well as PCTR as shown above. This proves that the HOMO, LUMO excitation efficiently ensures both charge separation and electron injection by moving the electron to the acceptor groups from donor moieties via π-linkers. Regarding optical properties, absorption maxima were observed at wavelengths of 462 and 463 nm for PCTR and PPR, respectively, and both molecules demonstrated λ_max_ coverage of 400 to 600 nm. The UV–vis absorption curves of pure cadmium sulfide NWs, which showed a shoulder at around λ_max_ 400 nm as well as an onset wavelength at around λ_max_ 508 nm, correspond to the band gap of 2.44 eV in the optical approach. When using PCTR and PPR with CdS, it was possible to observe absorption maxima at wavelengths of 495 and 499 nm, maintaining coverage from 400 to 600 nm. After characterization, compounds PPR and PCTR were evaluated as donor components in 1D-CdS nanowire sensitizers (CdS-NW). Photovoltaic studies reveal photovoltaic conversion efficiencies of the dye-sensitized CdS-NW PPR and PCTR of 0.226 and 0.288%, respectively. Based on the CV study, the calculated LUMO values of PPR and PCTR are −2.21 and −2.25 eV, values which are very close to the CB value of the CdS. The PCTR contains more efficient charge injection of photogenerated electrons than PPR, which might be the reason behind the enhancement in the solar cell parameters (*J*_SC_ = 1.013 mA ·cm^–2^, *V*_oc_ = 0.615 V) of the device containing PCTR molecules. The DFT studies reveal that PCTR molecules are located over oxygens of coumarin and carboxylic acid, which indicates itself as an electron-rich region that is also responsible for improving the solar cell performance of the device.^88^

## 3 Studies of Different π-Bridges

The π-bridge connects the donor and the acceptor, facilitating ICT. Consequently, the prototypical structure of metal-free organic sensitizers is represented as a donor-π-bridge-acceptor (D-π-A). Molecular engineering on the variety of donors and π-bridges has contributed to the obtainment of many sensitizers with good photovoltaic performance.^89^ In addition, double donors or acceptors were introduced into the sensitizer molecular structure. Coumarin-based D-π-A (donor-π-acceptor) compounds have significant advantages such as cost-effective molecule design, doubling frequency, structural flexibility, and short response time.^90,91^

**Scheme 5:**
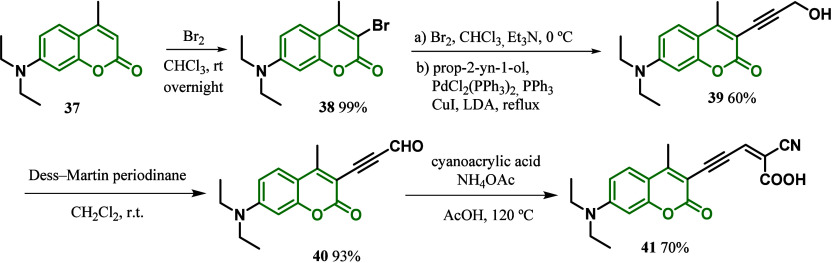
Synthesis of Coumarin Dye with an Ethynyl Group as a π-Spacer

In 2015, Torres and co-workers^92^ synthesized a coumarin **41** with an ethynyl group as a π-spacer unit for DSSCs. This is the first report of an alkyne π-conjugated bridge in coumarin dyes for DSSCs. More specifically, a 7-diethylamino-coumarin donating group and the cyanoacrylic acid electron acceptor are linked by a triple bond. According to the synthesis shown in Scheme 5, **41** was obtained via bromination of coumarin **37** at the C3 position, followed by a Sonogashira-Hagihara cross-coupling reaction and a subsequential selective oxidation using a Dess-Martin reagent. The last step was the Knoevenagel condensation of aldehyde **40** with cyanoacrylic acid to afford **41** in 70% yield. According to quantum chemical calculations, the HOMO is a *p* orbital that is delocalized throughout the molecule but mostly found over the coumarin and 7-diethylamino donor moieties. The LUMO is an orbital predominantly located across the cyanoacrylic acid acceptor group, but the alkyne π-bridge and coumarin core both contribute to the orbital. The power conversion efficiency of **41**-DSSC was 2.2% (*J*_SC_ = 6.11 mA·cm^2^) with a *V*_oc_ value of 547 mV. These results were equivalent to 68% of the device efficiency of the N719-based device fabricated similarly for comparison. According to EIS analysis, the performance of the **41** devices is not limited by charge recombination at the nanocrystalline TiO_2_/dye/redox electrolyte interface. The authors mentioned that a coadsorbent, such as stearic acid or deoxycholic acid, can be used to efficiently minimize dye aggregation, which is required to improve the performance of the **41** DSSCs.^92^

**Scheme 6:**
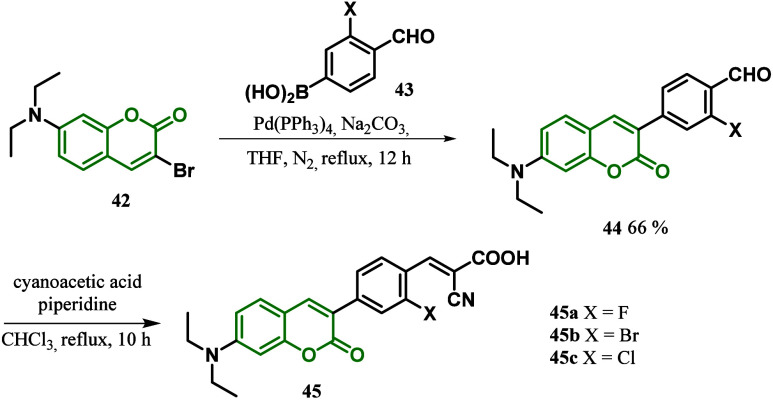
Synthesis of Coumarin Dyes **45a**–**c** with an ortho-Substituted Phenyl Group as a π-Spacer Unit

The 7-diethylamino coumarin donor moiety was also used by Vekariya and co-workers in 2017, who developed DSSCs with a new series of dyes having different halide groups on the ortho-position of substituted phenyl π-bridges. The dyes were synthesized using a 3-bromo-7-diethylaminocoumarin **42** and halogen-substituted phenyl boronic acids **43a**–**c** as starting materials. After the coupling reaction, intermediate **44** was condensed with cyanoacetic acid in the presence of piperidine to afford compounds **45a**–**c** (Scheme 6). The authors mentioned that the different halides in dye affected the photophysical and electronic properties of the corresponding DSSCs. According to the optimized geometry by theoretical calculations, **45a** has a planar structure and the HOMO levels are located on the coumarin donor moiety and strongly dispersed over the π-bridge. Moreover, **45b** and **44** showed HOMO levels mainly located on the coumarin moiety and slightly diffused into the phenyl π-bridge. For all dyes, the LUMO levels were mainly located on the acceptor group and π-bridges. The photovoltaic study showed superior photocurrent density for **45a** (*J*_SC_ = 10.4 mA·cm^2^) in comparison to those of **45b** (*J*_SC_ = 8.3 mA·cm^2^) and **45c** (*J*_SC_ = 7.5 mA·cm^2^), which was explained by the stronger electron-withdrawing capacity of the fluorine atom compared to chlorine and bromine. This electronic property of fluorine also explains the higher efficiency of **45a** (η = 5.2%) compared to that of **45b** (η = 4.1%) and **45c** (η = 3.5%), which might be associated with the loss of planarity and less molecular charge transfer upon introduction of Cl and Br atoms.^93^

**Scheme 7:**
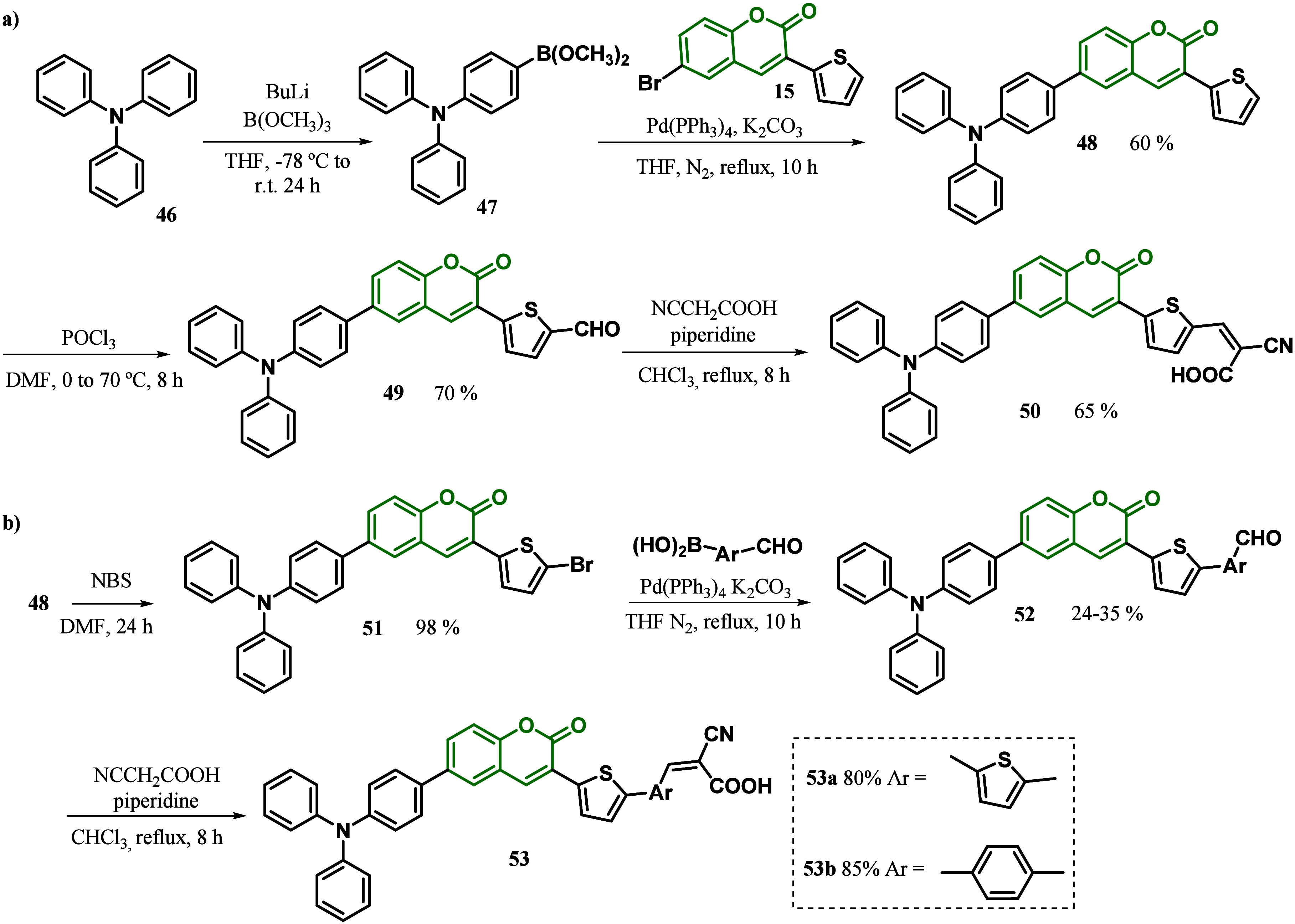
Synthesis of Coumarin Dyes a) with Thiophene π-Bridge **50** and b) with Bithiophene and Phenylthiophene π-Bridges **53a**–**b**

Another example using cyanoacrilic acid as an electron acceptor in the coumarin-based DSSCs was developed by He and co-workers.^94^ The authors designed three sensitizers with a D-D-π-A (donor-donor-π-bridge-acceptor) system, using a triphenylamine donor, a coumarin as an auxiliary donor, thiophene, bithiophene, and a phenylthiophene as a π-bridge and the cyanoacrylic acid acceptor (Scheme 7). The synthesis of **50** started with the conversion of the triphenylamine bromide **46** to the corresponding organic borate **47**, followed by the coupling with 6-bromo-3-thienylcoumarin **15** to give the intermediate **48**. Subsequently, an aldehyde was introduced with POCl_3_ and then condensed with cyanoacetic acid in the presence of piperidine (Scheme 7a). The synthesis of **53** is initiated with the bromination of **48**, followed by a second Suzuki coupling reaction with aldehydes and Knoevanagel condensation with cyanoacetic acid (Schema 7b). The optical spectra of the dyes in solution showed that their ICT peaks occur at 400–479 nm and the π–π* electronic excitation peaks at 340–350 nm. The addition of another electron-rich thiophene in **53a** exhibits the best absorption spectrum in solution, with maximum absorption wavelengths (λ_max_) at 343, 350, and 340 nm for **50** and **53a**–**b**, respectively. However, the best photoelectric conversion efficiency of 4.99% was achieved for **53c** (*J*_SC_ = 9.17 mA·cm^2^, *V*_OC_ = 0.70 V), as explained by the phenylthiophene π-bridge favoring the antiaggregation of the dye and enhancing the electron injection efficiency.^94^

**Figure 3:**
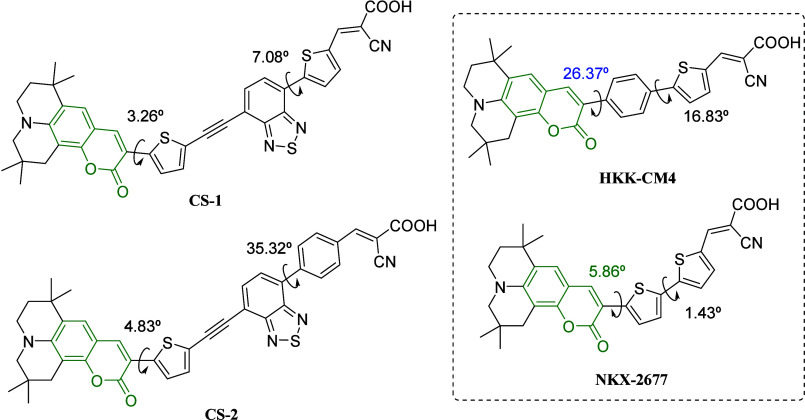
Coumarin is sensitized with a D-π-A-π-A system.

In the same year, Feng and co-workers^95^ developed DSSCs with an additional π-bridge between coumarin and the auxiliary acceptor in a D-π-A-π-A system. With this purpose, a thienyl ethyne group was introduced between coumarin (donor) and benzothiadiazole (acceptor) units as an additional π-bridge, improving the planarity of the donor-to-auxiliary acceptor part (Figure 3). Moreover, the authors studied the π-bridge segment between benzothiadiazole and the acceptor from the thienyl unit in **CS-1** to the benzyl unit in **CS-2**. In contrast to HKK-CM4, the dihedral angles between the coumarin and thienyl planes in **CS-1** and **CS-2** were determined to be 3.26 and 4.83°, respectively. In these cases, the introduction of an additional π-conjugation segment may efficiently smooth the ICT processes from coumarin to the auxiliary acceptor. In addition, the use of the large steric hindrance group in **CS-2** increased the dihedral angle between benzothiadiazole and the benzyl unit (35.32°) in comparison to **CS-1** (7.08°). As a result, **CS-1** showed higher molecular planarity and a better ICT process. According to DFT calculations, the HOMO orbital was delocalized throughout the coumarin and thiophene core, whereas the LUMO orbital was predominantly located across the benzothiazole, aromatic π-bridge, and cyanoacrylic acid acceptor group. Besides the excellent planarity observed in CS-1, their narrowest HOMO–LUMO band gap caused inefficient charge injection from LUMO to the conduction band of TiO_2_. As a result, the photovoltaic studies revealed that **CS-2** exhibited superior light-harvesting capability, with a maximum IPC value of 78% (470 nm), whereas the **CS-1**-based DSSC reached only 25% (470 nm). These results agreed with the highest short-circuit photocurrent density of **CS-2** (*J*_SC_ = 16.38 mA/cm^2^) in comparison to **CS-1** (*J*_SC_ = 5.09 mA/cm^2^). Moreover, **CS-2** also showed a high power conversion efficiency and a high open-circuit photovoltage, with η = 8.08% and *V*_oc_ = 694 mV, in comparison to **CS-1** (η = 2.20% and *V*_oc_ = 588 mV). According to the authors, the better photovoltaic performance of a D-π-A-π-A system in comparison to D-π-A dye NKX-2677^96^ demonstrates that planarity is important for the DSSCs.

**Scheme 8:**
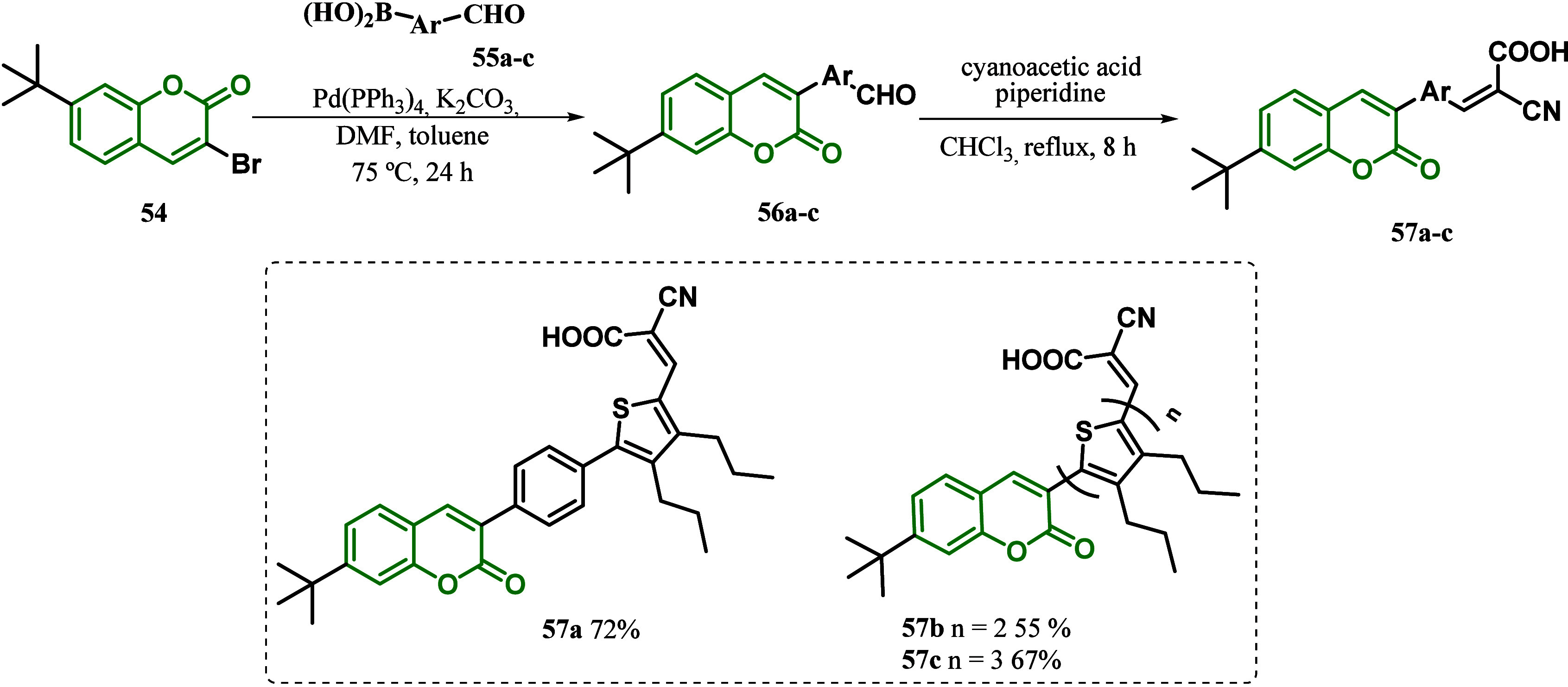
Synthesis of Coumarin Dye with a Thiophene Moiety-Based π-Bridge

In 2018, the Vekariya group^97^ also reported DSSCs with different numbers of thiophene moieties as a π-bridge in a D-π-A structure concept. The coumarin dyes were synthesized via a coupling reaction of 3-bromo-7-diethylaminocoumarin **54** and different boronic acids **55a**–**c**, followed by the condensation of intermediate **56** with cyanoacetic acid in the presence of piperidine to afford compounds **57a**–**c** (Scheme 8). According to the UV–visible spectra of the dyes in solution, increasing the thiophene π-bridge has a large influence on the absorption maximum (λ_max_) values of 449, 412, and 357 nm for **57c**, **57b**, and **57a**, respectively. In terms of the molar extinction coefficient (ε), **57c** has the highest value of 34666 M^–1^ cm^–1^. Additionally, when the thiophene bridge increased, the HOMO levels of synthesized dyes showed a little upshift, in which **57c** had a low band gap. Moreover, the π-bridge had an influence on the photovoltaic studies, in which the **57c** dye generated the highest efficiency at 6.02% compared to **57a** (η = 5.58%) and **57b** (η = 5.77%). The increasing thiophene bridge also leads to the short circuit current observed by the *J*_SC_ values of 15.04, 14.12, and 12.83 mA·cm^2^ for **57c**, **57b**, and **57a**, respectively. Besides that, the stability test showed that all dyes were stable after 500 h (under 100 mW·cm^2^ light illumination), showing just a slight reduction of the *J*_SC_ value. The authors conducted a DFT study to correlate the thiophene-bridge moieties with the photophysical properties of the dyes. The results indicated that the HOMO level is found in the coumarin group and the thiophene bridge while increasing the number of bridge moiety units did not affect the structural geometry or planarity.

**Scheme 9:**
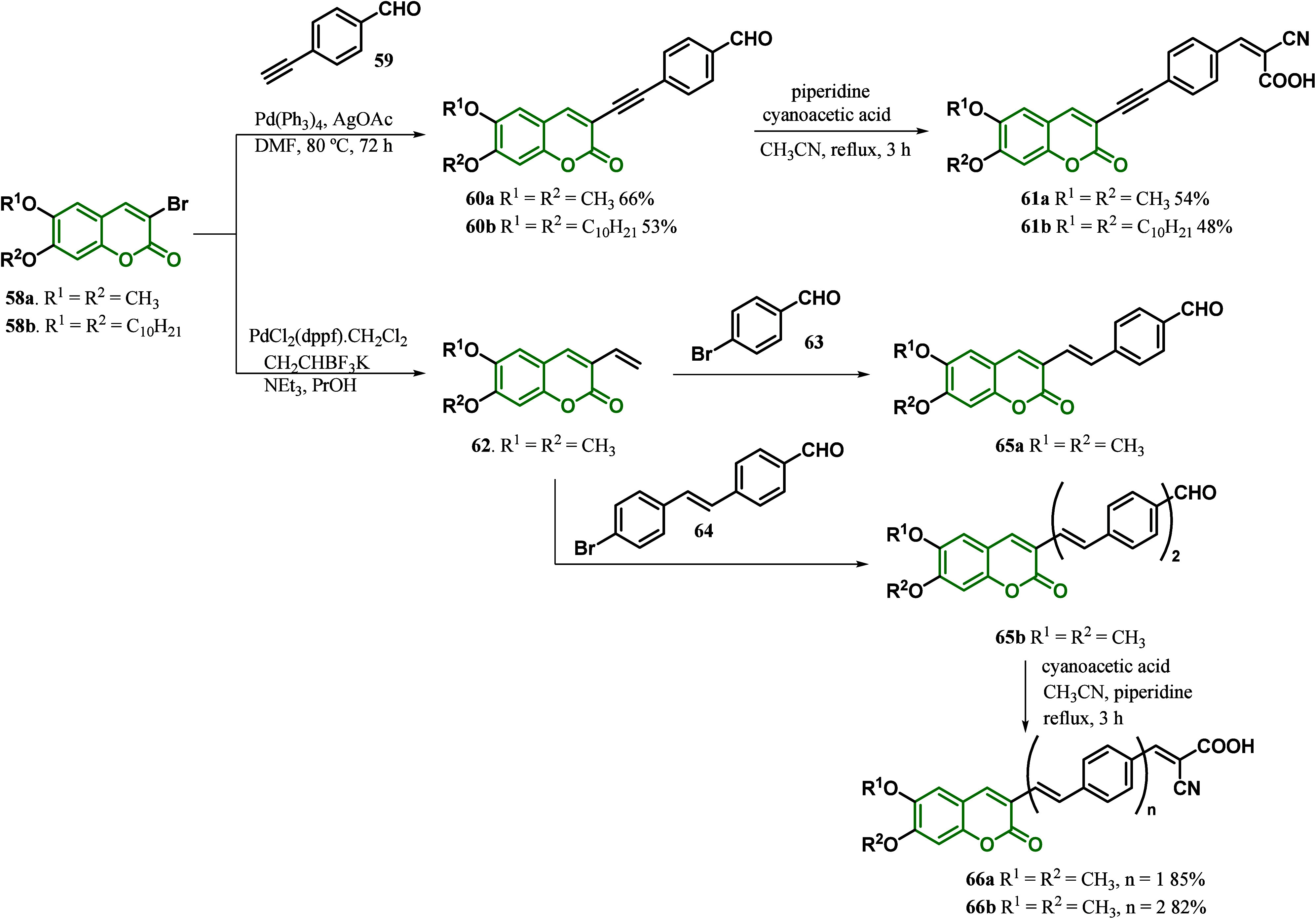
Synthesis of Styryl- and Phenylethynyl-Based Coumarin Dyes

In the same year, Martins and co-workers^98^ developed a styryl and phenylethynyl spacer in a coumarin-based dye for DSSCs, comparing the effect of double and triple bonds as π-bridges. The synthesis of the dyes was evaluated by using esculetin as a starting material. The 3-ethynyl coumarins **61a**–**b** were obtained via a Sonogashira reaction of coumarin **58** with 4-ethynyl benzaldehyde **59**, followed by the Knoevanagel condensation with cyanoacetic acid. On the other hand, for the 3-styryl coumarin derivatives **64a**–**b**, the first step was a Suzuki-Miyaura coupling, followed by a regioselective Heck arylation to afford the intermediates **65a**–**b**, which was subjected to a Knoevanagel condensation with cyanoacetic acid (Scheme 9). Dyes **61** and **66** were obtained as a mixture of the *Z* and *E* stereoisomers. The studies demonstrated that substitutions at the C6 and C7 positions of the coumarin core do not affect the photophysical properties of these dyes. However, the effect of the substitution of a double-bond spacer for a triple-bond spacer could be observed when comparing **61a** to **66a**, in which **66a** showed lower values of quantum yield. According to the authors, increasing the linearity imposed by the triple bond led to lower HOMO energy levels and improved the molecule’s charge transfer character. Moreover, the photovoltaic performance of the dyes revealed that for styryl derivatives **66a**–**b**, increasing conjugation does not account for any improvement in the DSSC performance. The authors justified the lower conversion efficiency of **66b** by the less effective electron injection to TiO_2_ and the isomerization of the second double bond, which compete for the deactivation of the excited state. However, when the styryl bridge of **66** is replaced with a more linear phenylethynyl spacer of **61**, the efficiency of the prepared DSSCs increases significantly, with η values of 2.0 and 1.37% for **66a** and **61a**, respectively. Interesting, as commonly observed in other chromophores for DSSC, this effect does not result from a decrease in the LUMO energy level. Instead, a dye regeneration rate may improve the performance of the cells, where substituing the double for triple bond enhances linearity, reduces the spatial separation between orbitals, and also leads to a marked decrease in the *E*_HOMO_ values.^98^

**Scheme 10:**
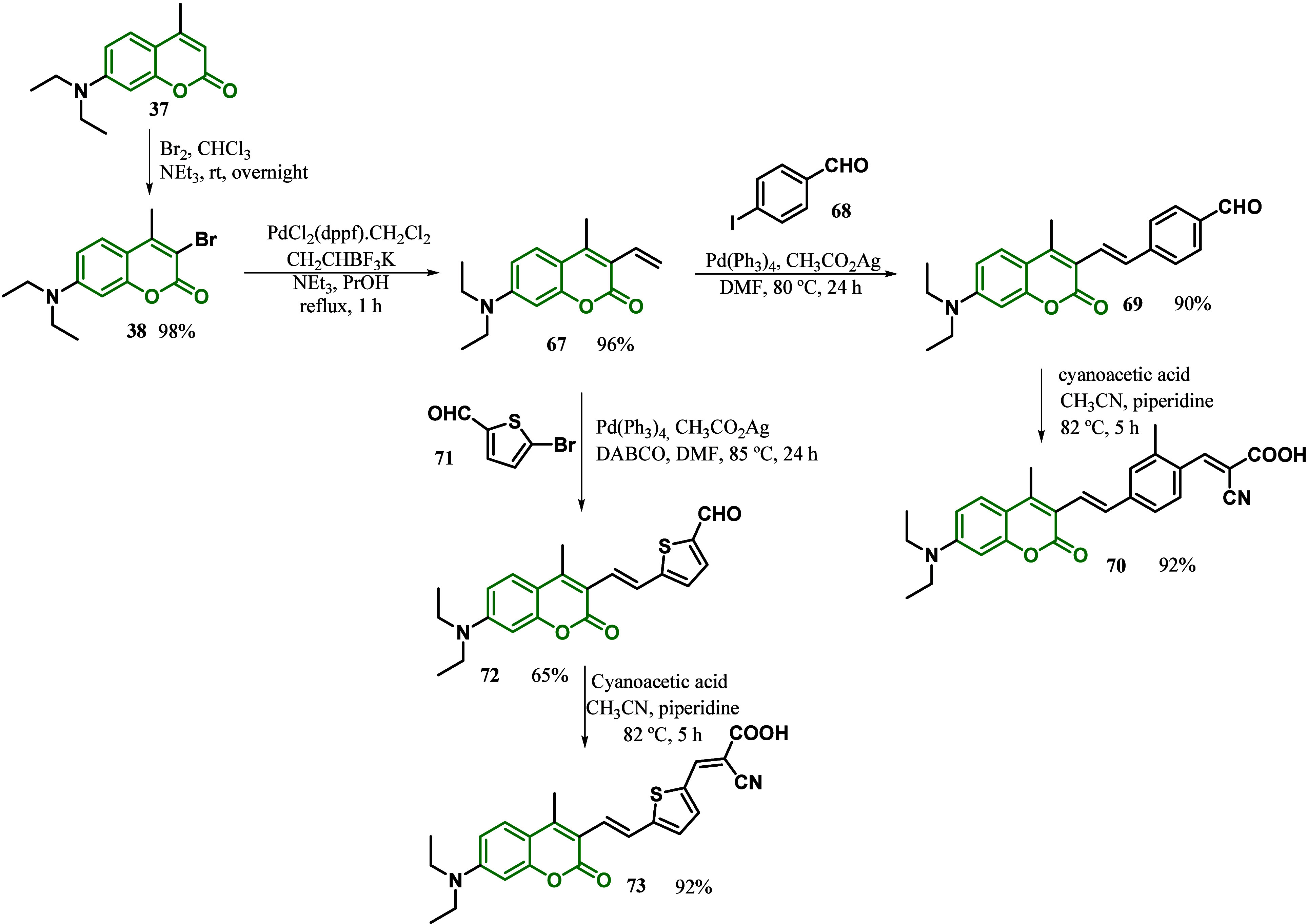
Synthesis of Coumarin Dyes **70** and **73** as Promising for DSSCs

In 2019, the same group developed the 7-(diethylamino)-4-methyl-3-vinylcoumarin as an effective synthetic strategy to produce coumarins as sensitizers for DSSCs.^99^ The synthesis started with a bromination reaction of low-cost coumarin **37**, followed by vinylation via Suzuki-Miyaura coupling to afford intermediate **67**. Next, a Heck reaction between the intermediate **67** and the halogenated aromatic aldehydes (4-iodobenzaldehyde) **68** and 5-bromothiophene-2-carbaldehyde **71**, followed by a Knoevenagel condensation with cyanoacetic acid afforded the photosensitizer coumarins for DSSCs, **70** and **69**, both in 92% isolated yield (Scheme 10). The studies demonstrated that the maximum absorption wavelengths were 452 and 496 nm for **70** and **73**, respectively. In comparison to the precursors, **69** and **72**, the final sensithizers **70** and **73** showed effective bathochromic shifts (17 and 50 nm, respectively), explained by the additional extension of the π-system. Moreover, due to the extension of the π-conjugated system, which is essential to an effective charge transfer process of the emissive excited state, both coumarin dyes exhibited high Stokes shifts describing the energy difference between the absorption and emission of light by the dye (84 and 73 nm for **70** and **73**, respectively). In terms of the UV absorption, the thiophene π-bridge sensitizer **73** exhibit a more pronounced red-shifted absorption than the π-bridge sensitizer **70** due to superior resonance energy of phenyl capable of delaying the electronic delocalization across the structure. According to the authors, the results suggested that these coumarin derivatives could be an interesting intermediate in synthesizing promising new photosensitizers for DSSCs.^99^

**Scheme 11:**
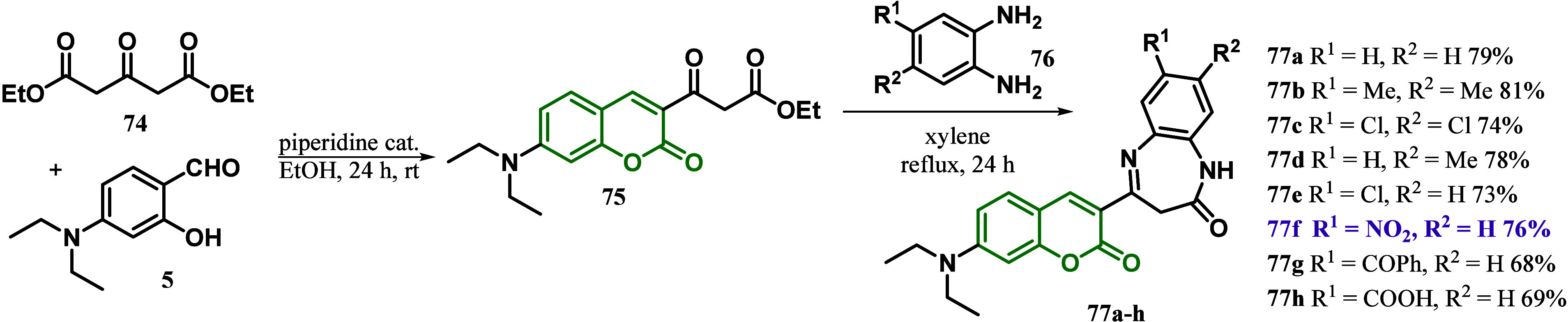
Synthesis of Hybrids Coumarin Benzodiazepines **77** as Interesting Precursors to Design Coumarin-Based D-π-A Sensitizers

In 2021, a coumarin-containing *N*,*N*-diethylamino group at the C7 position was also used by Gümüş to synthesize new dyes.^100^ The synthesis of the coumarin-based dyes was evaluated in two steps: First, the Knoevenagel reaction of diethyl 1,3-acetone-dicarboxylate **74** with 4-(diethylamino)-2-hydroxybenzaldehyde **5** afforded coumarin **75**, which was subsequently submitted to a cyclocondensation reaction with different *o*-phenylenediamine derivatives in PTSA catalysis (*p*-toluene sulfonic acid), as seen in Scheme 11. The authors mentioned that **77f** could be a precursor to designing coumarin-based D-π-A sensitizers since the λ_max_ for the π–π* electronic transition was observed at the highest values of between 447 and 461 nm in the experimental UV–vis studies. This could be expected due to the positive charge of the nitrogen atom in the nitro group, expanding the electron charge transfer.^100^

**Scheme 12:**
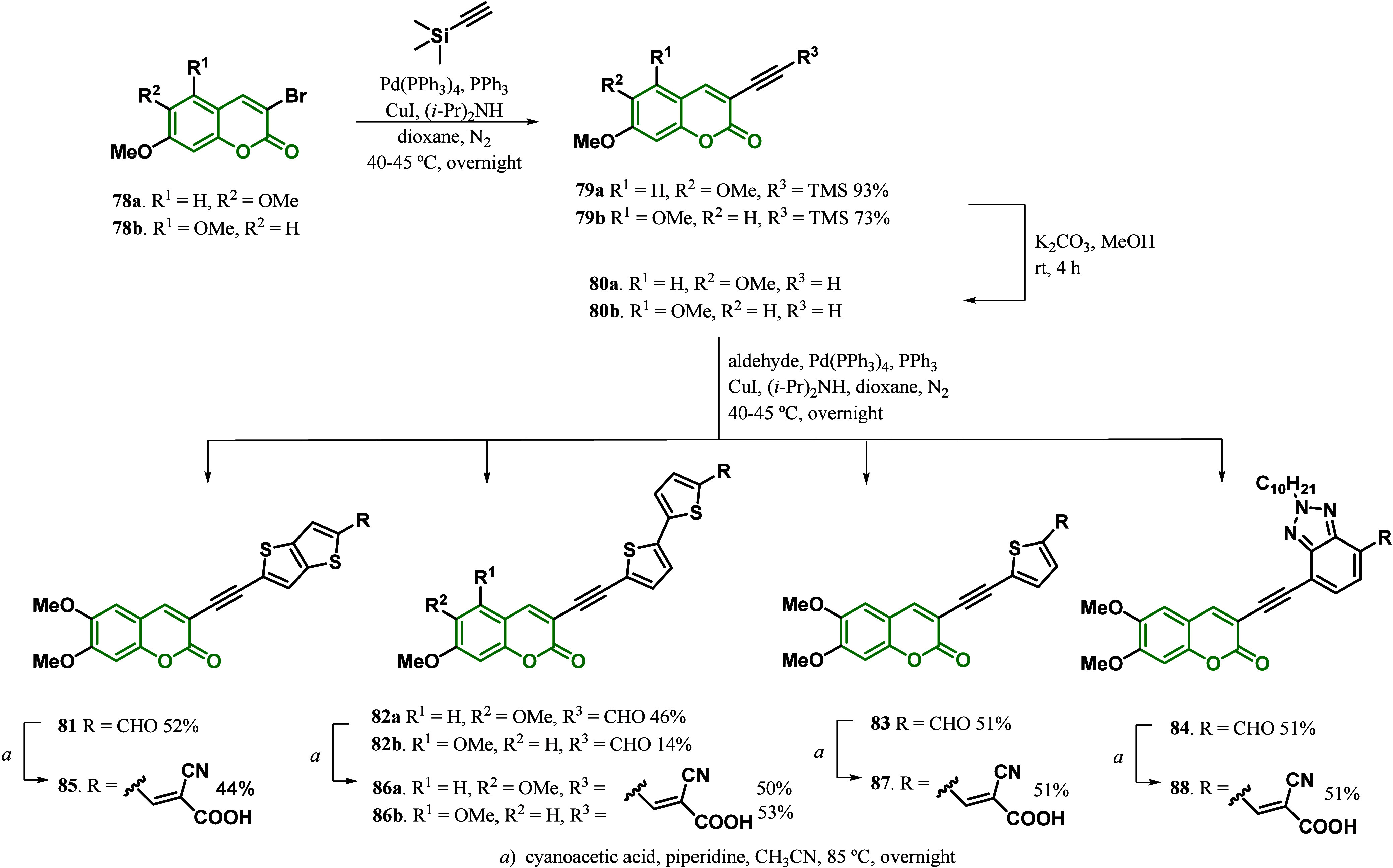
Synthesis of Mono- and Bisthiophenes, Thieno[3,2-*b*]thiophene, and Benzotriazole Coumarin-Based Dyes **85**, **86a**, **86b**, **87**, and **88**

Sarrato and co-workers^101^ prepared a set of 3-ethynylaryl coumarin dyes with mono- and bithiophenes, thieno[3,2-*b*]thiophene, and benzotriazoles for DSSCs. The synthetic approach was initiated with the brominated coumarin **78**, which was subjected to Sonogashira coupling with ethynyltrimethylsilane, followed by the removal of the trimethylsilyl group employing potassium carbonate in methanol. The intermediate **80** was then subjected to a second Sonogashira coupling reaction with the corresponding brominated aldehydes to insert the corresponding η-bridges moiety. Finally, Knoevanagel condensation of the aldehyde with cyanoacrylic acid in acetonitrile afforded the desired dyes in 14–53% yields (Scheme 12). The absorption spectra of the dyes showed λ_max_ values in the order of **87** = **88** < **86a** = **86b** < **85** with values of 375, 380, and 386 nm, respectively, which was explained by the strong electron-drawing nature of the cyanoacetic acid-substituted benzotriazole or thienyl units, resulting in high ground-state dipole moments. The theoretical calculations demonstrated that the HOMO orbital densities were generally spread across the entire molecule, whereas LUMO showed a decrease in electron density on the coumarin moieties and an increase in the benzotriazole or thienyl units. Moreover, coumarin **85** demonstrated the best photovoltaic performance, with an efficiency of 2% and the highest values of *V*_oc_ and *V*_max_, 367 and 256 mV, respectively. According to the authors, this could be explained by the planarity present in the fused thiophene ring, which is compared to **86a** (η = 0.95%) and **83** (η = 1.07%) that contain a 2,2′-bithiophene group and a thiophene group as a π-bridge, respectively. On the other hand, the comparison of dyes **86a** and **86b** allowed the evaluation of the effect of substituent position on the coumarin moiety, whose efficiency values (0.95 vs 1.78%, respectively) suggested that position **5** (when compared to **83**) is a superior choice for donor units in coumarin dyes. Dye **88**, which showed the worst *J*_SC_ value (4.4 mA/cm^2^) of all dyes and an efficiency of 1.13%, could be attributed to the benzotriazole moiety’s bulky alkyl chain, which will help suppress charge recombination between the semiconductor and the oxidized redox shuttle and affect decreasing the system’s planarity, reducing electron delocalization, and lowering the ICT character in the transition.^101^

## 4 Studies with Different Acceptor/Anchoring Groups

DSSCs necessitate the incorporation of one or more substituents within the molecules of their dyes, serving as anchoring entities to facilitate adsorption generally on a metal oxide substrate. This adsorption mechanism establishes a pathway for electron injection, a crucial process that initiates electrical transport within the DSSC circuit.^102^ A comprehension of the structural characteristics of diverse DSSC anchors and the exploration of novel anchoring agents constitute indispensable elements for advancing enhanced DSSC technologies. There are various types of anchor groups such as phosphonic acid, tetracyanate, perylene dicarboxylic acid anhydride, catechol, hydroxamate, sulfonic acid, boronic acid, salicylic acid, nitro, acetylacetonate, tetrazole, rhodanine, and aldehyde groups, but traditionally, carboxylic acid and cyanoacrylic acid groups have been conventionally utilized as dye anchors in DSSCs.^74,102−108^ In recent years, the emergence of innovative anchor groups has significantly expanded the repertoire of materials available for DSSC dyes.^109,110^ The associated physical and chemical characteristics of these novel anchors induce intriguing effects at the interface between the dye and metal oxide, contributing to the ongoing evolution and enhancement of DSSCs.

**Figure 4:**
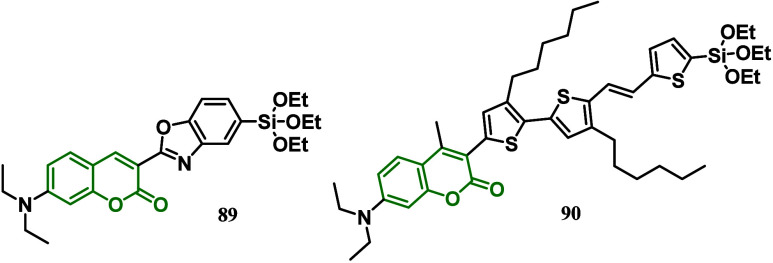
Silyl-anchor coumarin dye **89** and alkoxysilyl coumarin dye **90**.

In 2013, Kakiage and co-workers^111^ fabricated a DSSC using a novel silyl-anchor coumarin dye **89** with photoelectrodes consisting of the Mg-doped TiO_2_ powder and electrolyte solutions containing the Br_3_^–^/Br^–^ redox mediator (Figure 4).^112^ The cells showed a much higher *V*_oc_ than the cell with the ordinary TiO_2_ photoelectrode and the I^–^/I^–3^ redox mediator and successfully exhibited a remarkably high open-circuit photovoltage of over 1.2 V. However, the expected photovoltage of the cell was estimated to be ∼1.5 V. Thus, in 2016, the same group developed a DSSC using a novel silyl-anchor coumarin dye with alkyl-chain substitute **90**, a Br^–^/Br^3–^ redox electrolyte solution containing water, and an Mg^2+^-doped anatase-TiO_2_ electrode which presented a better efficiency than the undoped one.^111^

In the synthetic alkoxysilyl coumarin dye **90**, the introduction of alkyl-chain substituents was performed by linking hexyl-thiophene rings to the coumarin moiety (Figure 4).^113,114^ A methyl group was also added to the coumarin moiety to prevent the coplanar arrangement of the coumarin moiety and the thiophene ring, which led to a heightening of the HOMO level of the dye through the extension of the coumarin π-system to the thiophene ring. As a consequence, the observed open-circuit voltage was higher than those of other types of solar cells and is comparable to that of a conventional dry cell. A dry cell is a common type of electrochemical cell used in portable batteries, such as AA, AAA, and others. It is called dry because it does not contain free-flowing liquids, in contrast to wet cell batteries, which use liquid solutions as electrolytes. According to the authors, the introduction of alkyl-chain substituents near the silyl-anchor moiety in the coumarin dye is responsible for improving the photovoltage by preventing the back electron transfer from the electrode to the redox electrolyte. Thus, the achievement of such a high photovoltage, which is owing to the surpassing property of a silyl-anchor dye as a sensitizing dye for DSSCs, represents a practical application in photovoltaic devices.

Coumarin-based azo dyes and pigments are also fluorescent materials that present distinctive photochemical and photophysical properties and are vastly useful in several applications, including DSSCs. Furthermore, heteroaromatic rings like thiophene and furan are familiar as they act as building blocks of the π-bridge in the push–pull system.^115^

**Scheme 13:**
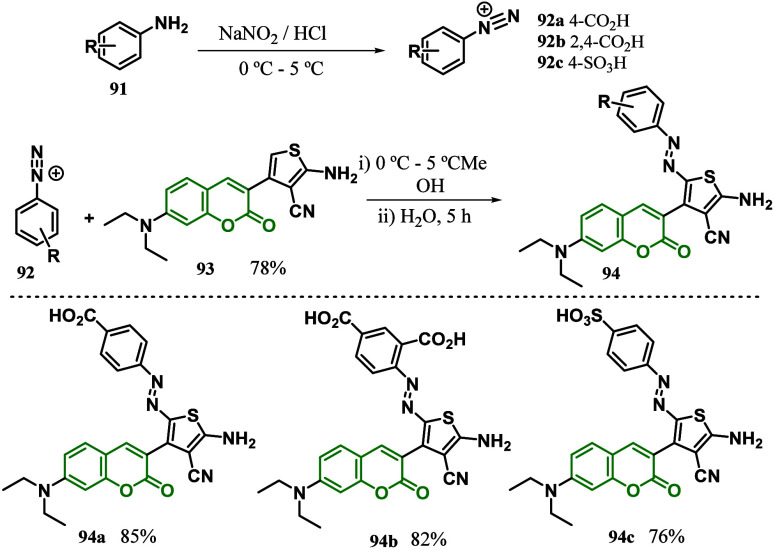
Synthetic Representation of Diazotization Used to Form Dyes

In 2020, Sekar’s group^116^ reported the synthesis of three coumarin-based D-π-azo-A colorants with −COOH and −SO_3_H anchoring groups, by Gewald reaction, for application in DSSC. The sensitizers were obtained from different anilines, where they were initially subjected to a diazotization reaction to obtain the corresponding azocompounds, which were then subjected to a coupling procedure against coumarin **93** to form the expected products **94a**–**c** (Scheme 13). The photophysical properties, thermal stability, and optical and electrochemical properties of the synthesized colorants were evaluated and subsequently utilized in DSSC to determine the photoconversion ability as a sensitizer. DFT computation was used to correlate the experimental findings of the synthesized dyes.The well-performing colorant **94a** exhibits a *J*_SC_ of ∼10.28 mA cm^–2^, with an open-circuit voltage, *V*_oc_, of ∼0.62 V. The electrochemical impedance spectroscopy (EIS) and electron lifetime (τ_e_) analysis showed effective performance with *R*_rec_ = 141.61 Ω and τ_e_ = 10.5 ms for the **94a** colorant, respectively. The **94a** that has the −COOH (an anchoring group) at the para position of the azo group with an FF of ∼68.7 exhibits the highest power PCE of 4.5 ± 0.2. Also, the electrophilicity index (ω), hyperhardness (Γ), and light-harvesting energy (LHE) were evaluated in DMF and demonstrated a similar trend with PCE (η). All of the sensitizers demonstrate an excellent nonlinear optical (NLO) response and display a direct relation with photovoltaic performance. In addition, the sensitizers exhibited affirmative α, β, and γ values in global hybrid functionals (B3LYP, BHHLYP) and a range-separated hybrid functional (CAM-B3LYP), revealing **94a** to be an excellent NLOphoric colorant.

## 5 Studies as a Cosensitizer

The term cosensitizer refers to a substance used alongside a primary sensitizing material to enhance the light absorption efficiency and its conversion into electricity. Cosensitizers typically work in conjunction with the main sensitizing dye in DSSCs. An effective strategy to achieve a broad absorption range across the visible region is the combination of different dyes that complement each other in their spectral characteristics.^117,118^

By mixing dyes with absorption at different wavelengths, it is possible to cover a broader range of the solar spectrum, thereby increasing the amount of light captured and converted into electricity. This technique has demonstrated remarkable potential, particularly in the field of DSSCs and perovskite devices, highlighting its versatility and promise in the evolution of photovoltaic technology.^118^

Recently, the introduction of π-bridge units such as thieno[3,2-*b*]indole (TI) instead of previous units such as thieno[3,2-*b*]benzothiophene (TBT) has shown significant improvements in DSSC efficiency. New organic sensitizers, such as SGT-136 and SGT-137, were designed to optimize the π-bridge capability, leading to a red shift in the absorption band and an increase in the energy levels of the highest occupied molecular orbital (HOMO),^119^ which enhances the short-circuit current density (*J*_SC_) and reduces charge recombination.^120^

Moreover, recent studies have demonstrated that the combination of different sensitizers can further increase the DSSC efficiency. For example, cosensitizing a fluorene-based dye, SGT-149, with a porphyrin-based dye, SGT-021, resulted in a power conversion efficiency (PCE) of 14.2%, one of the highest values ever reported for organic DSSCs.^120^ This improvement was attributed to the complementary spectral absorption of both dyes, enabling a broader solar spectrum coverage and a significant increase in light harvesting.^120^

Porphyrin-based sensitizers, such as SGT-021, have also shown great promise in extending the absorption into the near-infrared (NIR) region. DSSCs based on SGT-021 achieved an efficiency of 12.11%, surpassing the reference dye SM315 (11.70%), due to enhanced light-harvesting ability and reduced charge recombination.^118^ When used in tandem with other complementary sensitizers, such as SGT-137, efficiency increased even further, reaching a record of 14.64%.^117,118^

These advancements demonstrate that combining different sensitizers, especially those utilizing new π-bridge units and the molecular engineering of electron-donating groups, can not only increase *J*_SC_ but also expand the spectral absorption range. This enables more efficient light capture and, consequently, a more effective energy conversion process. The molecular engineering of TI-based and porphyrin-based sensitizers, with their enhanced electronic properties, opens new possibilities for the development of high-efficiency DSSCs.^117−123^

**Figure 5:**
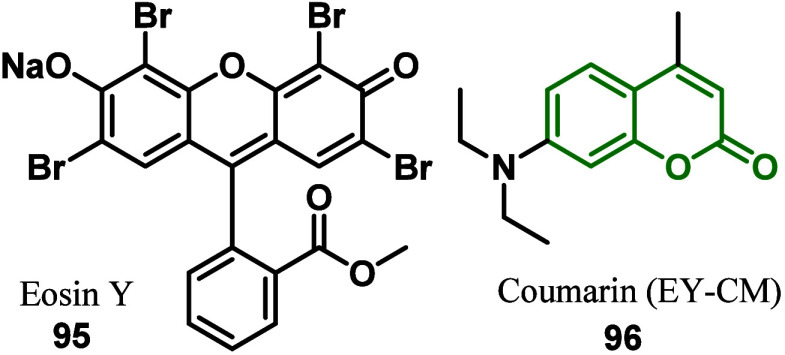
Cosensitizer composed of dyes Eosin Y (**95**) and Coumarin EY-CM (**96**).

In 2023, Subalakshmi and co-workers^124^ reported the development of high-performance DSSC (Figure 5). The structure of the device was formed by a metal-free organic dye cosensitizer and the hybrid photoanode. The cosensitizer was composed of Eosin Y **95** and coumarin (**96**, EY-CM) dyes, and the hybrid photoanode was constructed with titanium dioxide nanocomposites anchored in graphene oxide (rGO-TiO_2_).

This choice of device was designed to incorporate rGO, which could help improve electron transport because the energy level is between the CBs of TiO_2_ and FTO. Furthermore, cosensitization of EY and CM could lead to increased photovoltaic efficiency because of lower levels of LUMO aligned in neighboring dye molecules achieving an increased lifetime of electron injection and a decreased lifetime of photocarrier recombination. Therefore, rGO-TiO_2_ nanocomposites were synthesized through hydrothermal growth methods in a one-pot reaction using TiO_2_ precursors. From the studies of electrical impedance characteristics, by carrying out impedance spectroscopy, to better investigate the synergistic effects of the rGO-TiO_2_ hybrid photoanode and the MFO cosensitizer and analyze the photovoltaic characteristics of the DSSC devices, it can be observed that the synthesized nanocomposites presented aggregates of rGO-TiO_2_ nanostructures and exhibited enhanced charge transport characteristics due to the anchoring of highly conductive rGO with semiconductor TiO_2_.

After obtaining it, studies demonstrated that rGO-TiO_2_ coupling led to the formation of shallow Ti^3+^ donor levels within the TiO_2_ band gap, resulting in both increased charge transport and decreased photoelectron recombination. Cosensitization of EY and CM dyes complementarily increased the rate of light absorption over a wide spectral range. Furthermore, the EY-CM cosensitized system allowed for increased electron transfer from the electrolyte to the photoanode. Notably, the improved dye/photoanode adsorption by −COOH of EY and the aligned LUMO levels of EY-CM helped increase the electron pathways. All of these virtues synergistically led to the increase of both the electron lifetime and the chemical capacitance, which could eventually improve the photovoltaic performance of the fabricated rGO-TiO_2_/EY-CM DSSC. The results suggested that the proposed device scheme (i.e., hybrid photoanode + cosensitizer) is of excellent utility for improving the photovoltaic performance of DSSCs.^124^

**Scheme 14:**
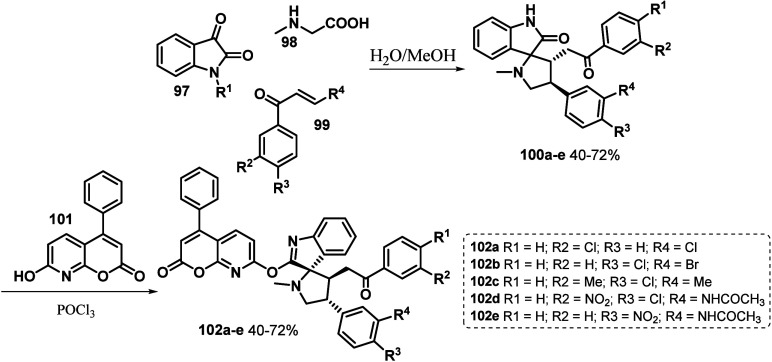
Synthesis of Spiro-Azacoumarin Dyes **102a**–**e**

The challenge in the continuous search for increased efficiency in the production of solar cells was one of the incentives for Attia and co-workers to invest in the development of new quality materials derived from perovskite, as it is already a component widely used in the manufacture of solar cells, forming a greater efficiency in light absorption and better photovoltaic behavior.^125^ In this way, different azacoumarin adducts were prepared using a series of previously synthesized spiroindolone moieties (Scheme 14). The dyes were obtained in good to moderate yields (40–72%).

The prepared compounds were subjected to quantum chemical calculations to investigate the molecular orbital parameters and study their quantitative structure–activity and structure–property relationships. The molecules showed good thermal stability with a decomposition temperature (5% weight loss) of 353 °C. The high thermal stability of SPCD prevents the deformation of the molecule morphology and degradation of the active layer of small molecular solar cells. The dye exhibits improved light-harvesting properties at wavelengths comparable to those of P3HT and an energy cascade compatible with P3HT and PCBM for both the LUMO and HOMO energy levels. Furthermore, an evaluation of the compounds prepared as antioxidants was carried out where the data revealed that the most effective concentration in the case of compounds **102a**–**e** is 200 ppm. The order of increasing inhibition efficiency of azacoumarin derivatives was classified as **102e** (16.5%) > **102d** (8.31%) > **102c** (1.39%) > **102b** (1.32%) > **102a** (1.27%), which was consistent with the order of greatest efficiency.

Studies of electro-optical properties revealed that sensitized dyes **102c**, **102d**, and **102e** are characterized in the visible light range and have good absorption and polarizability. Finally, to confirm the experimental data, quantum calculations based on DFT were carried out, which were able to provide insights into the structural and electronic characteristics of organic molecules, and according to the determined values, the order of efficiency of the dyes was **102e** > **102d** > **102c** > **102b** > **102a**, confirming the experimental results obtained. The authors then concluded that these materials are effective for renewable energy technology, that is, in DSSCs.^125^

**Scheme 15:**
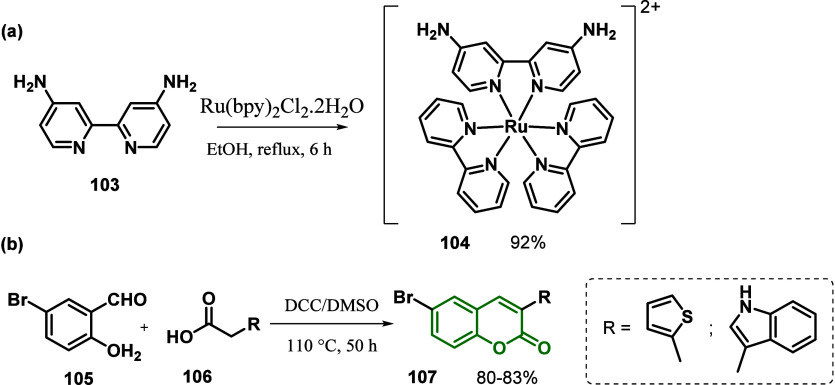
Syntheses of a) Ru(II)-Based Sensitizer **104** Containing Ligand 4,4′-Diamino-2,2′-bipyridine (dabpy) and b) Cosensitizers Derived from Coumarin

Athanas and co-workers^126^ reported a cosensitizer using coumarin derivatives and demonstrated that it is a powerful technique for PCE enhancement. In this work, the authors synthesized Ru(II)-based sensitizer **104** containing the ligand 4,4′-diamino-2,2′-bipyridine (dabpy) cosensitized with an electron donor–acceptor coumarin containing thiophene moieties (CT) and indole (CI) (Scheme 15). All compounds were obtained in excellent yields (80–83%) after purification.

The prepared compounds were evaluated for their photophysical and electrochemical properties, confirming their structures and interactions. Furthermore, a photovoltaic performance study of DSSCs was carried out where the authors synthesized a Ru(II)-based complex containing −NH_2_ anchoring group as a promising photosensitizer for highly efficient DSSC (5.44%). The photovoltaic performance might have been improved by cosensitization with coumarin cosensitizers of the electron donor–acceptor type containing thiophene and indole moieties (7.09 and 6.34%, respectively). The increased efficiency was attributed to the introduction of coumarin with increased solar energy absorption and HOMO–LUMO energy levels, decreasing dye aggregation, and charge recombination. Finally, they performed electrochemical impedance spectroscopy (EIS) tests to understand the kinetics within the electrode/electrolyte interface. RDAB1 shows a shorter lifetime (1.4 ms) compared to those of **104** + CT and **104** + CI. Both have almost the same lifetime value, i.e., 7.6 and 7.2 ms, respectively. The authors were able to conclude that increasing the electron lifetime increases efficiency.^126^

**Figure 6:**
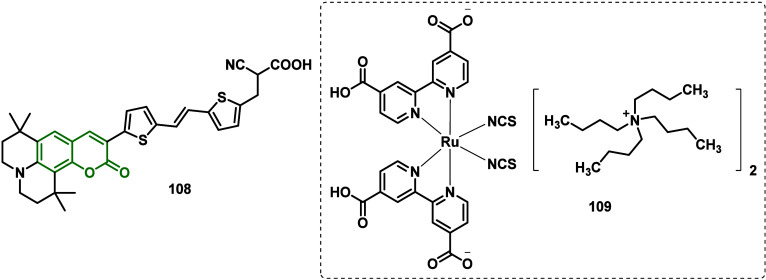
Molecular structures of coumarin **108** and complex **109**.

In 2015, Elangovan and co-workers,^127^ inspired by the group’s previous work, reported the continuation of the cosensitization study of two TiO_2_–Nb_2_O_5_ core/shell sensitizers with Ta_2_O_5_ scattering layer photoanode structures using two placements: complex **109** and dye coumarin **108** (Figure 6) and CdS/CdSe QDs and coumarin dye for application in DSSCs.

These structures and morphologies of TNPs, TNRs, and core/shell TNPRs/Nb_2_O_5_ were characterized and confirmed by XRD, TEM, and SEM. The PCE and electrochemical processes at the photoanode/dye/electrolyte interface were investigated by a photocurrent–voltage (*J*–*V*) study and electronic impedance spectroscopy (EIS) analysis, respectively. In the results obtained by the authors, it was noted that the PCE of cosensitizing DSSCs based on **109**, **108**, and CdS/CdSe QDs were 8.2, 6.9, and 4.5%, respectively. Thus, DSSCs with cosensitizers showed higher photocurrents than those of DSSCs with the corresponding individual sensitizers. Among the DSSCs, DSSC-5 made with **109**/**108** cosensitizer provided the highest PCE of 9.9% due to the highest values of *J*_SC_ = 21 mA/cm^2^, *V*_oc_ = 710 mV, and FF = 67%. The PCE of DSSCs made with **109**/**108** cosensitizer had the following order: DSSC-5 > DSSC-6 > DSSC 7 > DSSC-1.

It can be seen that the DSSC-9 with QDs/coumarin offered better photovoltaic characteristics, *J*_SC_ = 19 mA/cm^2^, *V*_oc_ = 700 mV, FF = 66%, and η = 8.7%, compared to the DSSCs made with the individual coumarin and QD sensitizers. The PCE of QDs/coumarin of cosensitized DSSCs follows the order DSSC-9 > DSSC-10 > DSSC-11. The cosensitized DSSCs exhibited enhanced PCE with the help of the synergistic stabilizing effect of both the organic dye and the inorganic dispersion layer in the presence of a corrosive electrolyte. Higher PCE values of DSSCs were associated with better adsorption of the sensitizer into the core/shell thin film and better absorption of UV/visible/infrared radiation from the source.

Studies of the electron transport properties of DSSCs were carried out by EIS. The Nyquist results for DSSCs 1–8 followed the order DSSC-3 > DSSC 4 > DSSC-2 > DSSC-1 > DSSC-7 > DSSC-6 > DSSC 5, which suggests that DSSC-5 contributed to the lower charge transfer resistance at the photoanode/dye/electrolyte interface. These factors effectively reduce charge recombination and improve cosensitization η DSSCs on **109**/**108** and QDs/**108**. Thus, cosensitization DSSC based on **109**/**108** and QDs/**108** produced a better PCE (Table 3). The results obtained in this study indicated that coumarin-derived cosensitizers are effective and can help improve the solar energy conversion efficiency.^127^

**Table 3:** Composition of Each Cosensitizing DSSC (1–11) and PCE Value

DSSC	Composition	PCE(η%)
DSSC-1	TNPRs/Nb_2_O_5_/Ta_2_O_5_/N719	8.2
DSSC-2	TNPRs/Nb_2_O_5_/Ta_2_O_5_/**108**	6.9
DSSC-3	TNPRs/Ta_2_O_5_/**109**	5.1
DSSC-4	TNPRs/Nb_2_O_5_/**109**	6.3
DSSC-5	TNPRs/Nb_2_O_5_/Ta_2_O_5_/**108**/**109**	9.9
DSSC-6	TNPRs/Nb_2_O_5_/**108**/Ta_2_O_5_/**109**	9.3
DSSC-7	TNPRs/Nb_2_O_5_/N719/Ta_2_O_5_/**108**	8.8
DSSC-8	TNPRs/Nb_2_O_5_/Ta_2_O_5_/QDs	4.5
DSSC-9	TNPRs/Nb_2_O_5_/Ta_2_O_5_/QDs/**108**	8.7
DSSC-10	TNPRs/Nb_2_O_5_/QDs/Ta_2_O_5_/**108**	8.0
DSSC-11	TNPRs/Nb_2_O_5_/**108**/Ta_2_O_5_/QDs	7.4

Table 4 provides a comprehensive summary of the main coumarin-based dyes used in the DSSCs described in this work, highlighting their characteristics and performances. Each analyzed dye is presented with a simplified representation or description of its molecular structure and its substituents. The quantitative data included in the table encompass the *J*_SC_, measured in mA/cm^2^, and the *V*_oc_, expressed in mV. The energy η is presented as a percentage, providing a clear view of the performance of each cell. Additionally, the maximum wavelength (λ_max_), in nm, and the maximum molar absorption coefficient, which assesses the dye’s absorption capacity, are highlighted. The oxidation potential is included to provide information about the dye’s stability and reactivity, while the Stokes shift is essential for understanding the difference between the absorption and emission wavelengths. The table also presents the emission wavelength (λ_em_), specified in nanometers, the electronic transition energy (E0–0), in volts (V), and the energy level of the lowest occupied molecular orbital (LUMO), also in volts (V). Finally, the fluorescence factor or fluorescent efficiency is included to indicate the dye’s ability to emit light after absorbing energy. This information allows for a direct and comparative analysis of the performance of each dye-sensitized solar cell, facilitating the evaluation of design strategies and the criteria guiding the selection of organic sensitizers in DSSCs.

**Table 4:**
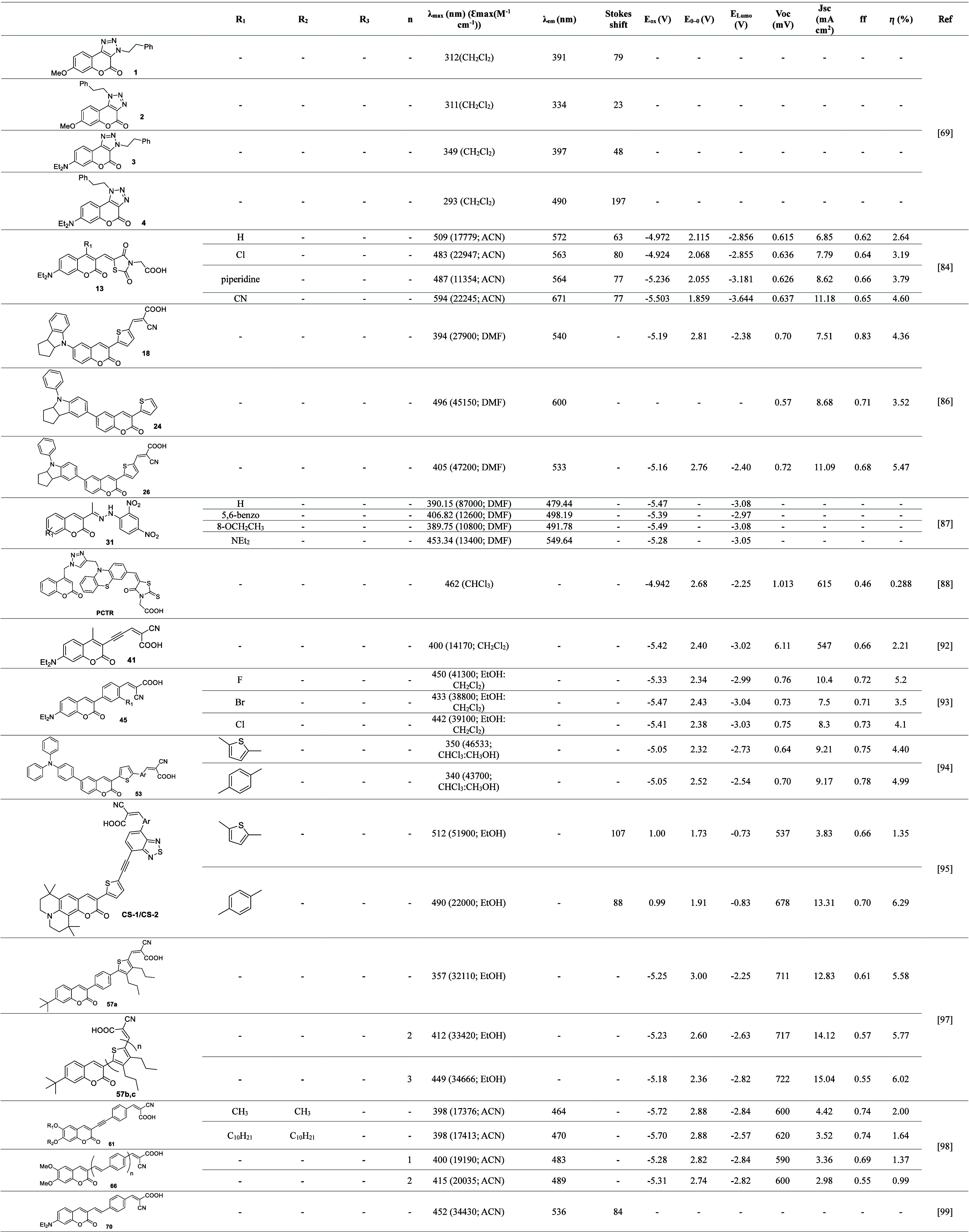
Properties and Performance of Coumarin-Based Dyes in DSSCs^a^

a *Molecules used as cosensitizers.

In the context of the synthesis of coumarin-based sensitizers, the pursuit of low-cost and highly efficient synthetic processes has been a prominent trend in the recent literature. Most of the studies presented demonstrate that it is possible to obtain these compounds through short and efficient synthetic routes with high yields using well-known and accessible reactions. For example, Knoevenagel condensation reactions, widely employed in forming the π-bridge between the donor and acceptor groups, stand out for their simplicity and efficiency, as they can be carried out under mild conditions with low-cost catalysts. Additionally, the functionalization of coumarins with simple donor groups, such as amines or phenols, can be achieved in just a few steps, reducing both the time and production costs. This approach is consistent with the donor structural engineering strategy presented by Zhou,^128^ where donor units such as hexyloxy-BFPA and BFPA were synthesized in only 4 or 7 steps, in contrast to the 11 steps required for the traditional hexyloxy-BPPA. Among all of the coumarin-based structures reviewed, the sensitizer CS-2, obtained by Feng and co-workers,^95^ stands out with the highest efficiency (6.29%). This result, combined with the low number of steps in its synthesis (5 steps), makes it one of the most promising structures for coumarin-based DSSCs evaluated in this review. The simplification of the synthetic process, coupled with the use of accessible raw materials, not only reduces costs but also maintains or even improves the photovoltaic efficiency of the sensitizers. Therefore, the synthesis of coumarin-based sensitizers through short and efficient routes represents a promising approach to the development of economically viable and sustainable DSSC, aligning with current demands for low-cost, high-performance solar technologies.

## 6 Conclusions

Faced with the growing demand for renewable and sustainable energy sources, DSSCs emerge as a promising alternative in photovoltaic energy, standing out for their efficiency, cost-effectiveness, and versatility. In this context, sensitizers play a key role in the efficient conversion of solar energy into electricity within these systems.This review article focused on the use of coumarins, a class of aromatic organic compounds, as a platform for sensitizers in DSSCs. We explore the synthesis of coumarins and their photophysical and electronic properties as well as their application in optimizing the energy conversion efficiency of DSSCs. Additionally, we examine molecular engineering strategies to enhance the properties of coumarins including structural modifications and combinations with other materials.

By addressing current challenges and prospects for developing DSSCs using coumarins as sensitizers, this review seeks to contribute to a more comprehensive and advanced understanding of this exciting area of solar energy research. With the dissemination of knowledge about the properties and applications of coumarins, discoveries and innovations are expected to boost the field of DSSCs, leading to significant advances in the efficiency and commercial viability of these devices.

In conclusion, faced with the growing demand for renewable and sustainable energy sources, DSSCs emerge as a promising alternative in photovoltaic energy, standing out for their efficiency, cost-effectiveness, and versatility. In this context, sensitizers play a key role in the efficient conversion of solar energy into electricity within these systems. A class of aromatic organic compounds that have been used as a platform for sensitizers in DSSCs are the coumarins, due to their photophysical and eletronic properties. This review article focused on the synthesis and application of coumarin derivatives in optimizing the energy conversion efficiency of DSSCs. Additionally, we examine molecular engineering strategies to enhance the properties of coumarins, including structural modifications and combinations with other materials. By addressing current challenges and prospects for developing DSSCs using coumarins as sensitizers, this review seeks to contribute to a more comprehensive and advanced understanding of this exciting area of solar energy research. With the dissemination of knowledge about the properties and applications of coumarins, discoveries and innovations are expected to boost the field of DSSCs, leading to significant advances in the efficiency and commercial viability of these devices
